# A comprehensive evaluation of Hippo pathway silencing in sarcomas

**DOI:** 10.18632/oncotarget.25824

**Published:** 2018-08-03

**Authors:** Nicole M. Merritt, Colleen A. Fullenkamp, Sarah L. Hall, Qining Qian, Chandni Desai, Jon Thomason, Allyn M. Lambertz, Adam J. Dupuy, Benjamin Darbro, Munir R. Tanas

**Affiliations:** ^1^ Department of Pathology, University of Iowa, Iowa City, IA, USA; ^2^ Department of Pediatrics, University of Iowa, Iowa City, IA, USA; ^3^ Department of Anatomy and Cell Biology, University of Iowa, Iowa City, IA, USA

**Keywords:** Hippo pathway, sarcoma, TAZ, YAP, epigenetics

## Abstract

TAZ and YAP are transcriptional coactivators negatively regulated by the Hippo pathway that have emerged as key oncoproteins in several cancers including sarcomas. We hypothesized that loss of expression of the Hippo kinases might be a mechanism of activating TAZ and YAP. By immunohistochemistry, TAZ/YAP activated clinical sarcoma samples demonstrated loss of MST1 (47%), MST2 (26%), LATS1 (19%), and LATS2 (27%). Western blot similarly demonstrated loss of MST1 (58%), MST2 (25%), and LATS2 (17%). Treatment with MG132 demonstrated an accumulation of MST2 in 25% of sarcoma cell lines, indicating that proteosomal degradation regulates MST2 expression. qRT-PCR in sarcoma cell lines demonstrated loss of expression of the Hippo kinases at the RNA level, most pronounced in *MST1* (42%) and *MST2* (25%). 5-azacytidine treatment in sarcoma cell lines modestly reversed expression of predominantly *MST1* (8%) and *MST2* (17%), indicating CpG island hypermethylation can silence expression of *MST1* and *MST2*. Trichostatin A treatment reversed expression of *MST1* (58%) and *MST2* (67%), indicating histone deacetylation also plays a role in silencing expression of *MST1* and *MST2*. Loss of expression of the Hippo kinases is frequent in sarcomas and is due to a variety of mechanisms including regulation at the post-translational level and epigenetic silencing.

## INTRODUCTION

TAZ (*WWTR1* is the gene) and YAP (*YAP1* is the gene) are developmentally important transcriptional coactivators [1, 2] that have emerged as central oncoproteins in a number of carcinomas including breast [3, 4], colon [5], liver [6], lung [7, 8], pancreas [9], and thyroid cancers [10]. TAZ (transcriptional coactivator with PDZ-binding motif) and YAP (yes associated protein) do not contain DNA binding domains of their own, and must complex with other transcription factors that contain DNA binding domains via their TEAD binding domain or WW domain [11–15]. The TEA domain (TEAD) family of transcription factors have been demonstrated to be the dominant transcription factors with regards to mediating the TAZ and YAP transcriptional programs [16, 17]. TAZ and YAP are negatively regulated by the Hippo pathway, a series of serine/threonine kinases including the STE20-like protein 1 and 2 (MST1/2) [18–20] and the large tumor suppressor 1 and 2 (LATS1/2) [21, 22]. The MOB1A/B [23] and Salvador proteins [20, 24] have been shown to form a scaffold for the above kinases. Cell confluence [2] and detachment [25] activate the Hippo pathway, causing LATS1/2 to phosphorylate TAZ and YAP on several serines, including serine 89 on TAZ and serine 127 on YAP. Phosphorylation of these serines leads to binding of 14-3-3 proteins, ultimately resulting in translocation of TAZ/YAP from the nucleus into the cytoplasm, where they undergo ubiquitin-mediated degradation [1, 2].

A number of different signal transduction pathways have been identified that modulate the activity of the Hippo kinases or TAZ and YAP directly. The Wnt signaling pathway has been shown to activate TAZ and YAP directly via their interaction with β-catenin [26, 27]. The PI3 kinase pathway activates TAZ by inhibiting glycogen synthase kinase-3 [28] and activates YAP by promoting the dissolution of the Hippo kinase signaling cascade [29]. G protein coupled receptors have been shown to activate TAZ and YAP by dampening activity of the LATS1 and LATS2 kinases [30–32]. This has led to a paradigm that TAZ and YAP are activated predominantly via cross-talk with other signal transduction pathways.

In contrast, primary lesions affecting the Hippo kinases have been rarely identified. Although TAZ and YAP have been shown to be activated oncoproteins in a number of carcinomas [33, 34] and sarcomas [35], genetic alterations are rare with the exception of the *WWTR1*-*CAMTA1* [36–38] and *YAP1*-*TFE3* gene fusions in epithelioid hemangioendothelioma (EHE) [39]. Mutations in the upstream Hippo kinases, *MST1*, *MST2*, *LATS1*, and *LATS2* have also been rare. Occasional mutations have been identified in the scaffolding proteins *MOB1A/B* [33]. *In silico* studies have suggested that copy number changes (deletions) of genes upstream of the Hippo kinases (e.g. *NF2*) may result inactivation of TAZ and YAP [40], however the frequency of these genomic alterations is incompletely understood. More recently, the Itch ubiquitin ligase has been shown to reduce expression of the LATS1 kinase [41, 42]. Scattered reports indicate the presence of promoter hypermethylation of several of the Hippo kinases, although evaluation of how promoter hypermethylation affects expression levels and functional activity of the kinases is incomplete [43–45]. In summary, although various lines of evidence indicate the presence of primary lesions of the Hippo pathway, their true frequency and significance is incompletely understood. To address this, we performed a comprehensive evaluation of expression of the Hippo kinases in sarcomas, a group of cancers that have recently been shown to harbor frequent activation of the TAZ and YAP oncoproteins [35].

## RESULTS

### Expression of the Hippo kinases is lost in TAZ/YAP activated clinical sarcoma samples

**Figure 1:**
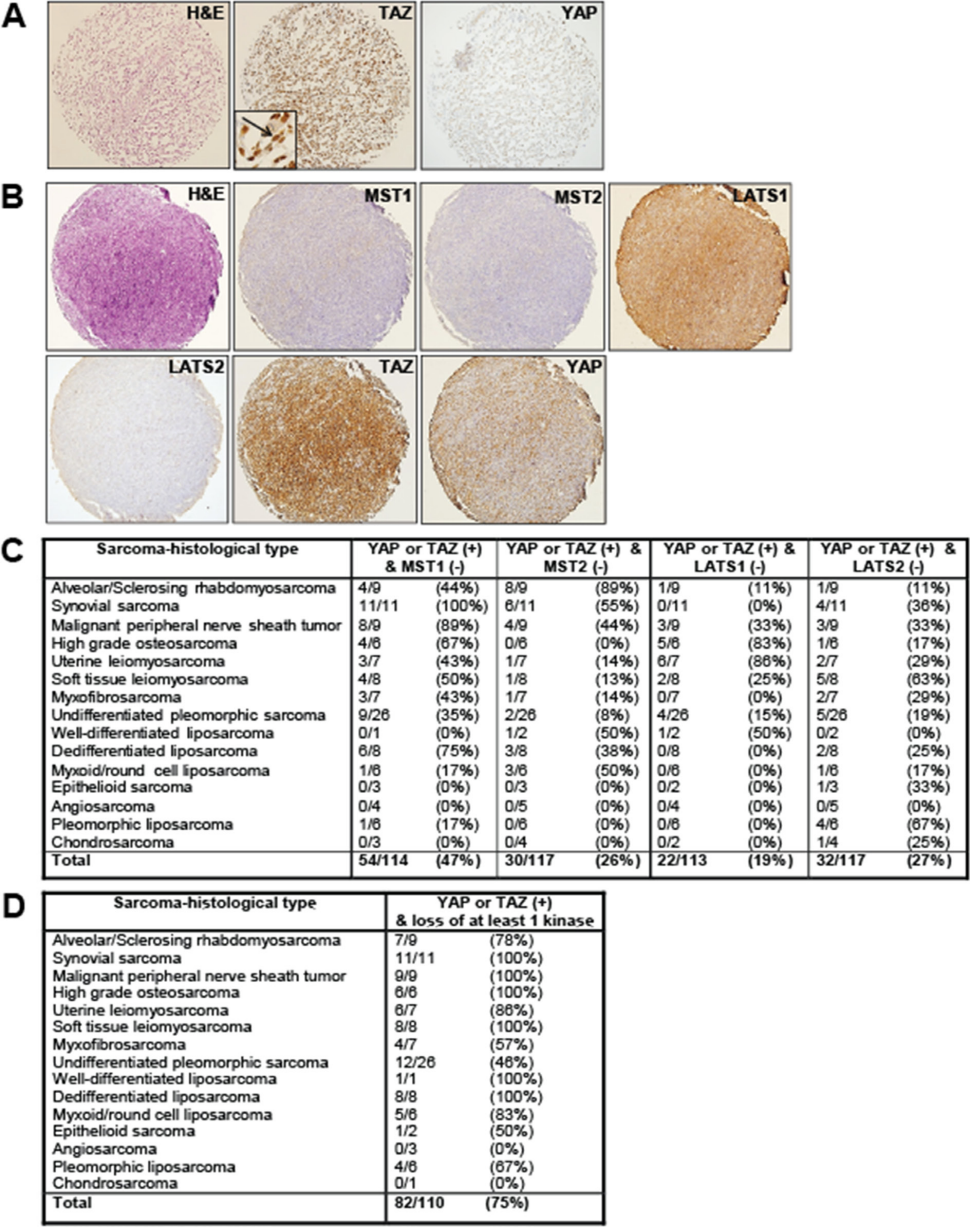
Expression of the Hippo kinases in clinical samples Immunohistochemical evaluation of MST1, MST2, LATS1, and LATS2 in a sarcoma tissue microarray. (**A**) Myxofibrosarcoma demonstrating diffuse and strong expression and nuclear localization (activation) of TAZ, and a lack of expression of YAP. (**B**) Synovial sarcoma demonstrating a lack of expression of MST1, MST2, and LATS2 in a sarcoma with activated TAZ and YAP. LATS1 expression is maintained. (**C**) Table demonstrating range of Hippo kinase loss in YAP or TAZ activated sarcomas. Loss of expression ranged from 19% (LATS1) to 47% (MST1). (**D**) Table demonstrating the frequency of loss of at least one Hippo kinase as a function of sarcoma histological type in sarcomas demonstrating activated TAZ or YAP.

We have recently demonstrated that TAZ and YAP are constitutively activated and located within the nucleus of the majority of sarcoma clinical samples. Evaluating expression of TAZ and YAP in multiple histological types of sarcoma revealed that approximately 50% of sarcoma clinical samples demonstrate activated YAP, while 66% demonstrate activated TAZ. (Figure 1A) [35]. Mutations within components of the Hippo pathway have reported to be rare [33]. Evaluation of 259 sarcomas in The Cancer Genome Atlas demonstrated a mutation rate ranging from 0% for *MST2* and *LATS1* to 0.8% for *LATS2* (Supplementary Figure 1A). Several lines of evidence indicate that silencing of the Hippo kinases is necessary for activation of TAZ and YAP. The TAZ-CAMTA1 fusion protein has been demonstrated to negate inhibition from the Hippo pathway [38]. Other lines of evidence indicate that the Hippo pathway is silenced secondarily via interactions with other pathways [26–29]. Some reports have addressed the possibility that the Hippo pathway is primarily silenced through promoter hypermethylation [43–45] or ubiquitin mediated degradation [41, 42], however this has not been investigated in a comprehensive manner.

One mechanism by which the Hippo pathway could be primarily silenced is through loss of expression of the Hippo kinases. To assess this possibility, we evaluated expression of the Hippo kinases in an unbiased way through immunohistochemistry for MST1, MST2, LATS1, and LATS2 on the tissue microarray (Figure 1B and 1C). Normal smooth muscle was utilized as a positive control (Supplementary Figure 1B). TAZ and YAP activated sarcomas, defined as sarcomas demonstrating intermediate level intensity of staining and greater than 70% of cells showing nuclear localization of TAZ or YAP were previously identified [38]. At least 113 TAZ/YAP activated sarcomas were available for evaluation. Within these TAZ/YAP activated sarcomas, 47% (54/114) demonstrated loss of MST1 expression, 26% (30/117) demonstrated loss of MST2 expression, 19% (22/113) of the sarcomas demonstrated loss of LATS1 expression, and 27% (32/117) of the sarcomas demonstrated loss of LATS2 expression (Figure 1C). Hippo kinase expression loss in all sarcomas (regardless of TAZ or YAP activation status) was similar. Approximately 49% of sarcomas (73/148) demonstrate loss of MST1, 31% of sarcomas (46/148) demonstrate loss of MST2, 23% of sarcomas (34/148) demonstrate loss of LATS1 expression, and 29% of sarcomas (45/153) demonstrate loss of LATS2 (Supplementary Figure 1C). The similar frequency of loss of the Hippo kinases in TAZ/YAP activated sarcomas versus all sarcomas regardless of TAZ/YAP activation status indicates that loss of the Hippo kinases is associated with TAZ/YAP activation the majority of the time.

We next determined the frequency at which at least one of the Hippo kinases was lost among a panel of 110 sarcomas. Approximately 75% of the sarcomas demonstrate a loss of expression of at least one of the Hippo kinases (Figure 1D). All of the synovial sarcomas, malignant peripheral nerve sheath tumors, high grade osteosarcomas, soft tissue leiomyosarcomas, well-differentiated liposarcoma, and dedifferentiated liposarcomas demonstrated loss of expression of at least one of the Hippo kinases. The frequency of loss of expression in the remaining sarcomas ranged from 46% (12/26) in undifferentiated pleomorphic sarcoma to 86% (6/7) in uterine leiomyosarcoma (Figure 1D). No angiosarcomas or chondrosarcomas were noted to exhibit loss of the Hippo kinases, but the sample sizes of these sarcomas were small.

We then looked at other combinations of loss of the Hippo kinases in YAP or TAZ activated sarcomas in an effort to ascertain what combinations of the Hippo kinases are required for activation of TAZ/YAP to occur. Loss of expression of MST1 and MST2 was identified in 15% (17/114) of sarcomas. Loss of expression of LATS1 and LATS2 was noted in 8% (9/113) of sarcomas. Loss of expression of all four Hippo kinases was identified in 1 of 110 (1%) of all sarcomas (Supplementary Figure 1D).

### Expression of the Hippo kinases is lost in TAZ/YAP activated sarcoma cell lines

**Figure 2:**
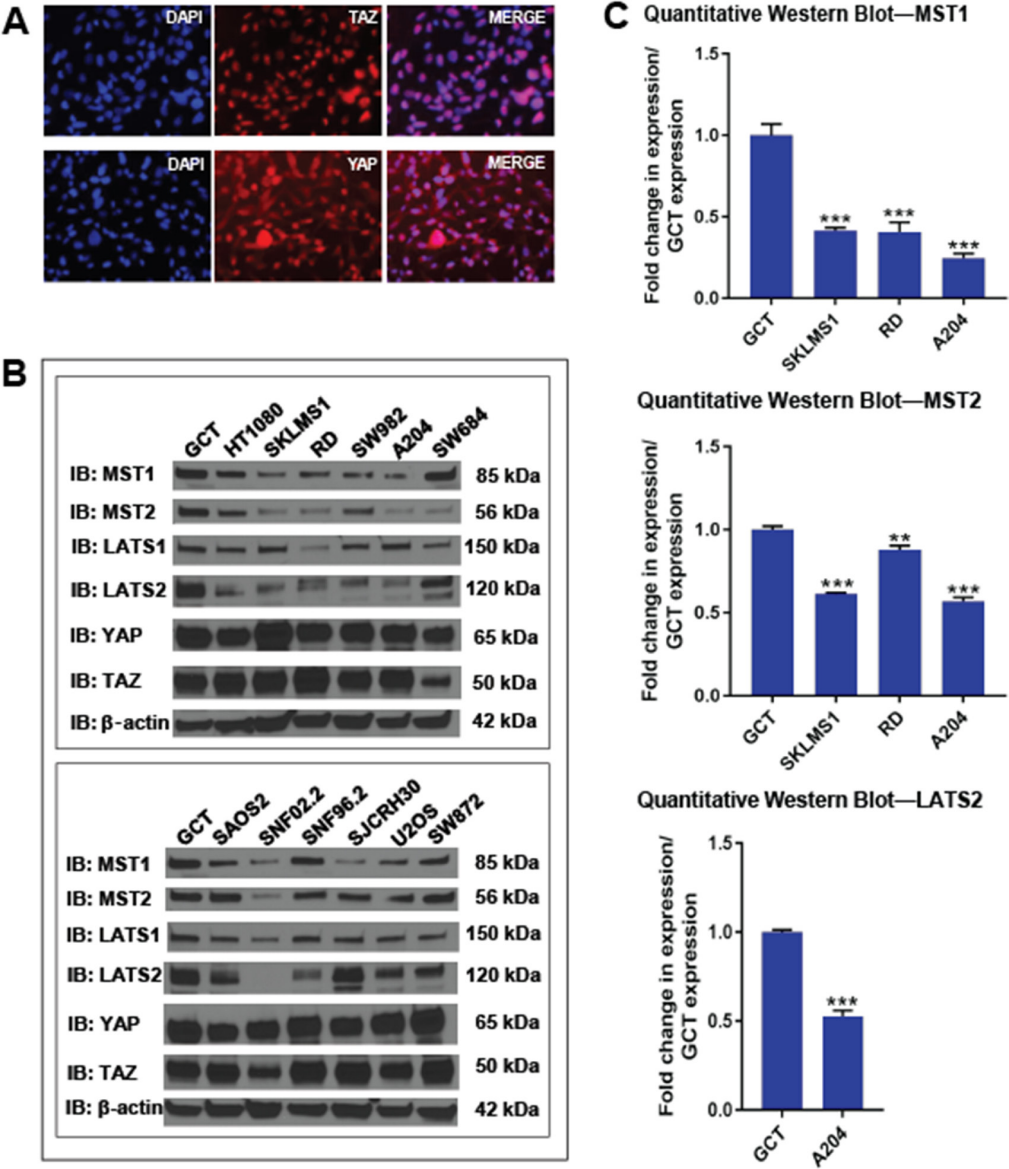
Expression of the Hippo kinases *in vitro* (**A**) Immunofluorescence demonstrating constitutive nuclear localization of TAZ and YAP in A204 cells grown to confluence. (**B**) Western blot demonstrating a loss of expression of Hippo kinases relative to GCT (giant cell tumor) cell line. (**C**) Quantitative western blot for MST1, MST2 and LATS2 (the most commonly lost kinases) in the SKLMS1, RD, and A204 cell lines and compared to GCT. The SKLMS1, RD, and A204 cell lines showed a stastically significant decrease in expression in MST1 and MST2 as compared to GCT. Of these three lines, only A204 demonstrated a significant decrease in expression of LATS2. Statistical significance determined by two-tailed *t*-test. ^*^ indicates *p* < 0.05; ^**^ indicates *p* < 0.01; ^***^ indicates *p* < 0.001.

As we have previously shown, TAZ and YAP are constitutively activated and located within the nucleus of sarcoma cell lines (Figure 2A, Supplementary Figures 2 and 3). Essentially all of the sarcoma cell lines assayed demonstrate nuclear localization of TAZ and YAP when grown to confluence (Supplementary Figures 2 and 3), consistent with a lack of negative regulation by the Hippo pathway. With the exception of MOB1A/B [33], mutations in the upstream Hippo kinases (especially MST1/2) have been reported to be extremely rare. Mutations in TAZ and YAP have also been shown to be extremely rare, with the exception of the *WWTR1*-*CAMTA1* and *YAP1*-*TFE3* gene fusions which are disease defining genetic alterations found in essentially all epithelioid hemangioendotheliomas [37–39]. In the majority of cancers, *WWTR1* and *YAP1* have been reported to lack mutations [33]. To confirm this is the case in sarcomas, we performed targeted PCR-based Sanger sequencing of 12 sarcoma cell lines and GCT (giant cell tumor) for the presence of mutations in the serines in TAZ (S66, 89, 117, and 311) [46] and YAP (S61, 109, 127, 164, and 381) [2] phosphorylated by LATS1/2. GCT is an immortalized, non-sarcoma mesenchymal cell line, and expression of the Hippo kinases appeared to be similar as compared to MCF10A, an immortalized but non-transformed cell line commonly utilized as a negative control epithelial cell line in the field [3, 47] (Supplementary Figure 4A). For this reason, this cell line was used as a control in the subsequent experiments. None of the 13 cell lines evaluated by Sanger sequencing demonstrated mutations in the above mentioned serine residues (Supplementary Figure 4B).

Expression levels of the Hippo kinases were then evaluated at the protein level by western blot. Qualitatively, expression of at least one of the Hippo kinases was reduced by approximately 2-fold in 10 of the 12 sarcoma cell lines (83%) compared to GCT (Figures 2B and 7A), closely approximating the 75% of clinical samples that had lost at least one of the Hippo kinases (Figure 1D). Quantitative loss of the MST1, MST2, and LATS2 kinases, the kinases whose expression was most commonly lost, was confirmed in a sampling of 3 cell lines, SKLMS1, RD, and A204 (Figure 2C and Supplementary Figure 5A).

**Figure 3:**
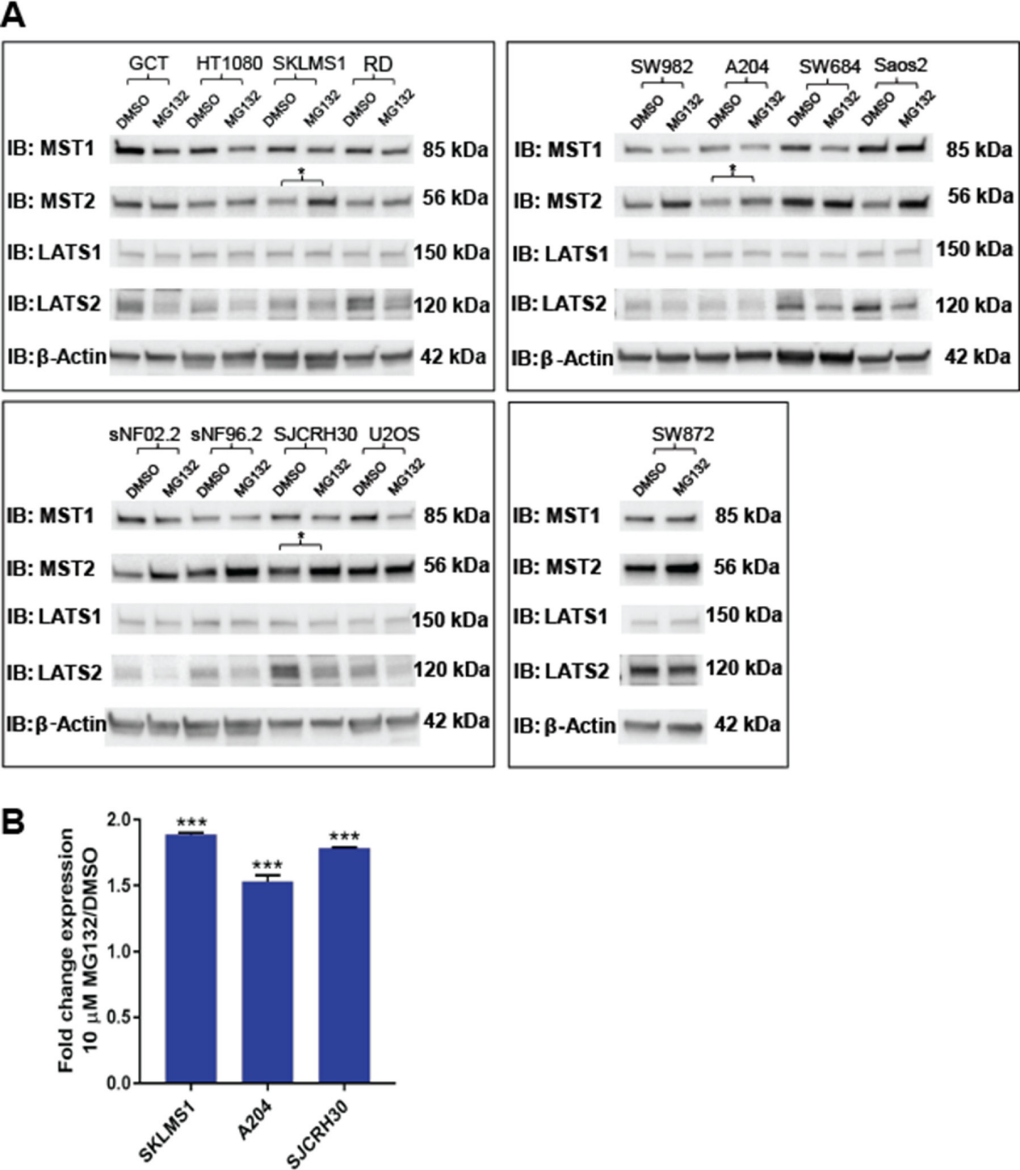
Evaluation of proteosomal degradation of the Hippo kinases *in vitro* Cell lines were treated with 10 μM MG132 for 12 hrs. A 1.5-fold or greater accumulation of protein was considered indicative of proteosomal degradation. (**A**) Western blot demonstrates accumulation of MST2, but not MST1, LATS1, or LATS2 with treatment with 10 μM MG132. This was validated quantitatively in 3 cell lines (SKLMS1, A204, and SJCRH30) in part (**B**). These three lines are indicated by an asterisk in part (A). Statistical significance determined by two-tailed *t*-test. ^*^ indicates *p* < 0.05; ^**^ indicates *p* < 0.01; ^***^ indicates *p* < 0.001.

Protein expression levels can be regulated at multiple levels, including altered protein turnover. To test the hypothesis that protein degradation could play a role in Hippo kinase expression, the 12 sarcoma cell lines were treated with 10 μM MG132 for 12 hours. After 12 hours, MG132 was shown to quantitatively increase expression of MST2 in 3 of the 12 cell lines (25%) (Figure 3 and Supplementary Figure 5B), indicating that enhanced proteosomal degradation leads to decreased Hippo kinase expression in sarcoma cell lines. None of the other Hippo kinases showed evidence of increased proteosomal degradation. In some cell lines in which MST2 expression increased with MG132 treatment, LATS2 expression was found to demonstrate the opposite relationship and decreased.

### Copy number changes (deletions) of the Hippo kinases are not a common event in sarcomas

**Figure 4:**
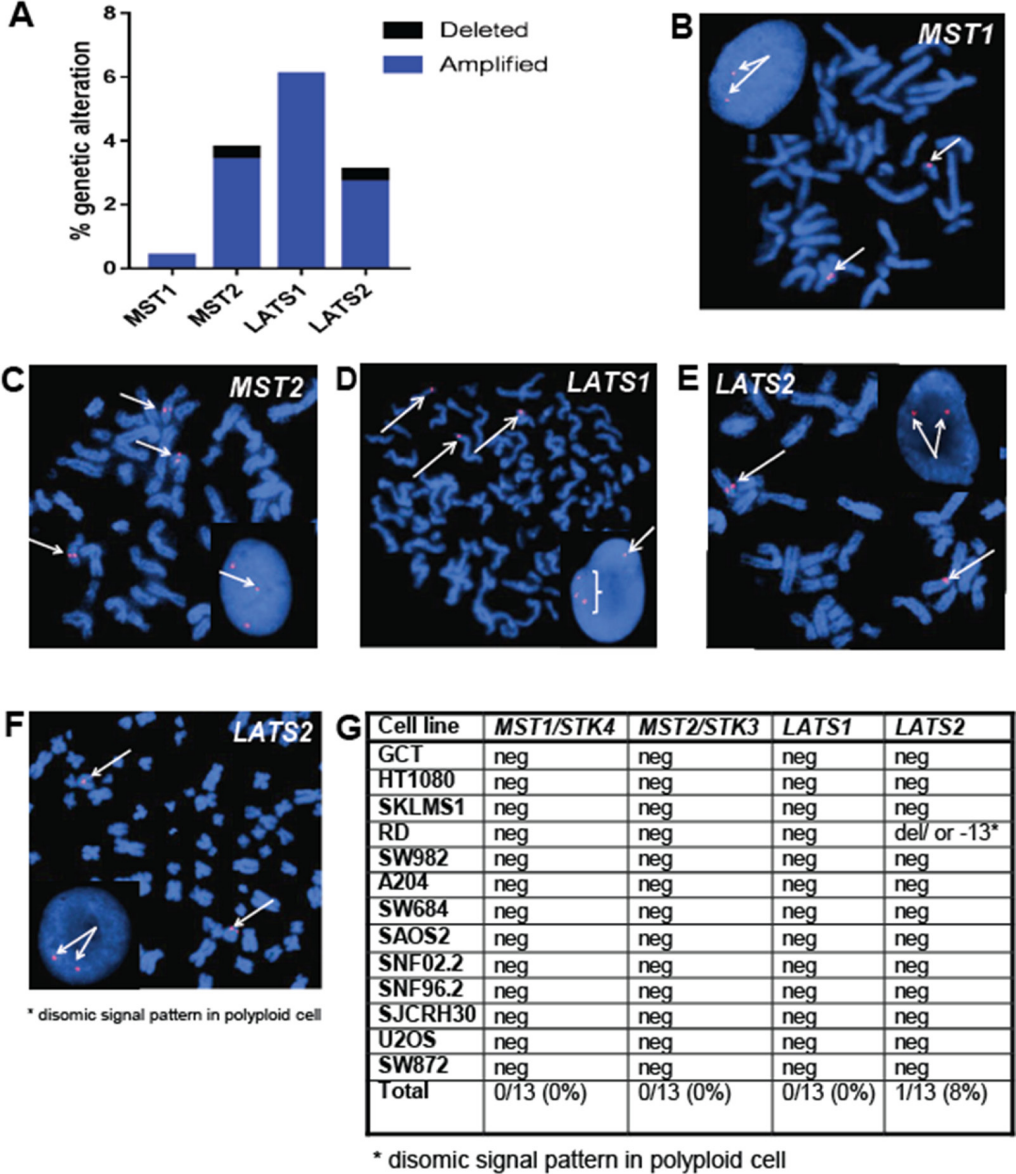
The Hippo kinases are rarely deleted in sarcomas (**A**) Evaluation of The Cancer Genome Atlas data set revealed less than 1% of sarcomas demonstrate deletions of *MST2* and *LATS2*. (**B**–**D**) Fluorescence *in situ* hybridization (FISH) utilizing fluorescent labeled BAC probes hybridizing to metaphase chromosomes and interphase nuclei (inset). (B) FISH probes for *MST1* (20q13) demonstrating two signals in metaphase chromosomes and two signals in the interphase nucleus in the A204 cell line, indicating no deletions of the *MST1* gene region are present. (C) FISH probes for *MST2* (8q22) demonstrating polyploidy, but no deletions of the *MST2* gene region. (D) FISH probes for *LATS1* (6q25) demonstrate polyploidy, but no deletions of the *LATS1* gene region (**E**) FISH probes for *LATS2* (13q12) demonstrate a normal disomic pattern in both the metaphase chromosomes and the interphase nucleus in the A204 cell line, indicating no deletions of the *LATS2* gene region (**F**) FISH for *LATS2* in the RD cell line, demonstrating disomic signal pattern in a polyploid cell. Although a deletion or loss of chromosome 13 is present, two copies of *LATS2* are still present. (**G**) Table summarizing FISH results. No deletions were observed in MST1, MST2, or LATS1. A deletion was noted in RD, however the cell line is polyploid, and two copies of LATS2 were still present.

To evaluate the possibility that loss of expression was occurring due to alterations at the genomic level, copy number changes/deletions were evaluated utilizing data from The Cancer Genome Atlas data set. Prior studies in sarcomas have highlighted *in silico* data emphasizing deletions in NF2 (merlin), a regulatory protein upstream of the Hippo pathway [40]. Potential genomic deletions in the Hippo kinases themselves have not been thoroughly investigated. *In silico* analysis demonstrated that 0.8% of sarcomas demonstrated genomic deletions of MST2 and LATS2 combined, indicating that deletions are not a common mechanism by which loss of expression of the Hippo kinases occurs (Figure 4A). To confirm the *in silico* data, we performed fluorescence *in situ* hybridization (FISH) for *MST1*, *MST2*, *LATS1*, and *LATS2* on cell lines (Figure 4B–4G).

No genomic deletions of the Hippo kinases were identified by FISH in the sarcoma cell lines, with the exception of the RD cell line. The RD cell line demonstrated a disomic signal for *LATS2* in a polyploid cell, indicating a deletion or loss of chromosome 13. Given that the *LATS2* copy number is still two, the contribution to LATS2 expression is unclear.

### Expression of *MST1* and *MST2* is reduced at the RNA level in sarcoma cell lines

**Figure 5:**
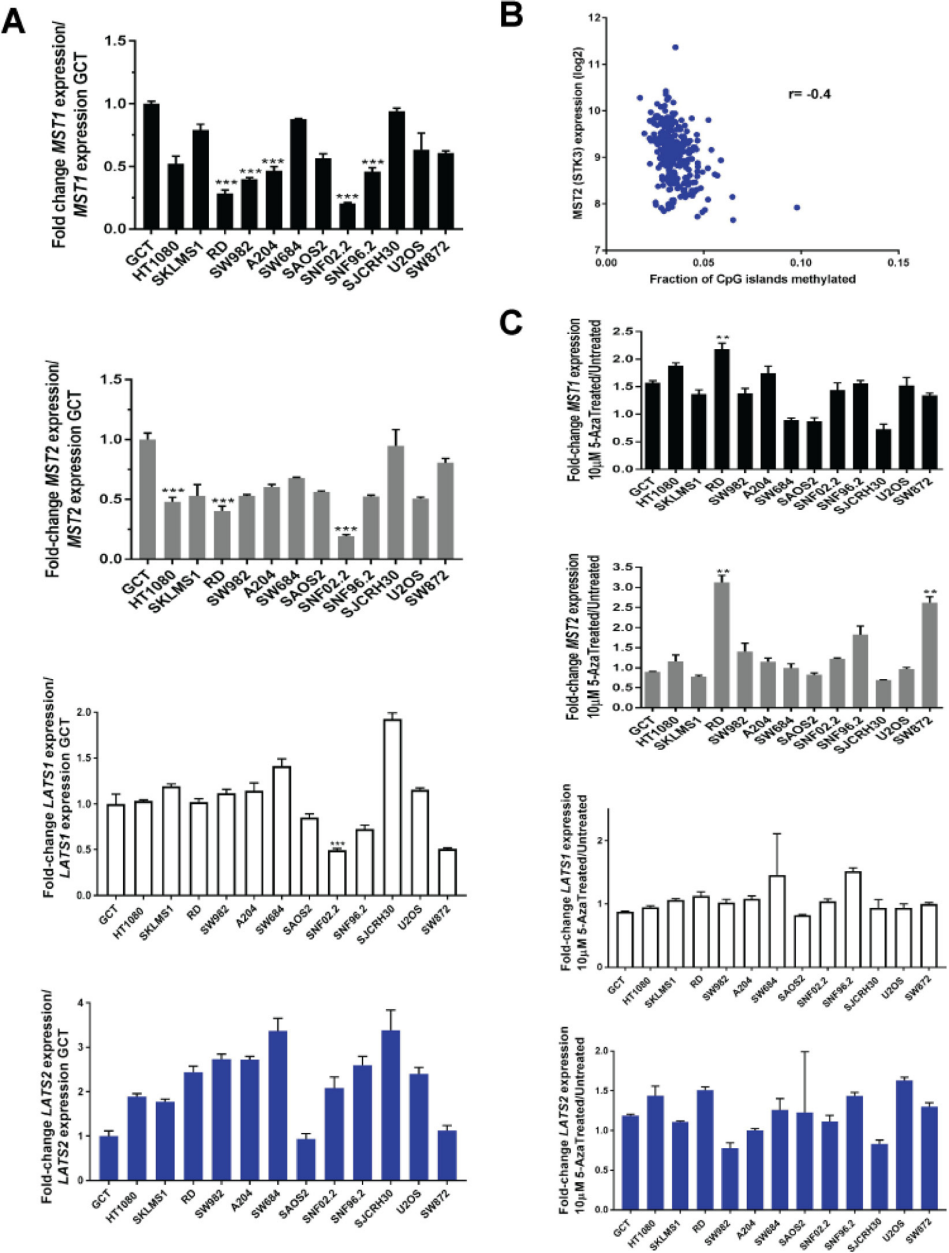
Loss of expression of the Hippo kinases at the RNA level and reconstitution with 5-azacytidine in sarcoma cell lines evaluated by quantitative RT-PCR Loss of expression was defined as a two-fold or greater decrease of expression of the Hippo kinases (0.5 fold decrease in expression as compared to GCT). Increase in expression was defined as a two-fold or greater increase in expression after treatment with 10 μM 5-azacytidine. (**A**) *MST1* expression is decreased at the RNA level in 5 of 12 sarcoma cell lines (42%). *MST2* expression is decreased in 3 of 12 sarcoma cell lines (25%). *LATS1* expression is decreased in 1 of 12 sarcoma cell lines (8%). No decrease in *LATS2* expression is detected (0%). (**B**) TCGA data demonstrating that *MST2* expression is inversely proportional to CpG island methylation (Spearman's correlation coefficient *r* = −0.4). (**C**) *MST1* expression is increased after treatment with 5-azacytidine in 1 of 12 cell lines (8%). *MST2* expression is increased after treatment in 2 of 12 cell lines (17%). Neither *LATS1* nor *LATS2* expression is increased in any (0%) of the cell lines after treatment with 5-azacytidine. Statistical analysis is shown for cell lines demonstrating a two-fold or greater decrease in expression. Statistical significance determined by two-tailed *t*-test. ^*^ indicates *p* < 0.05; ^**^ indicates *p* < 0.01; ^***^ indicates *p* < 0.001.

Since genomic alterations/deletions of the Hippo kinases was not responsible for decreased expression of the Hippo kinases, and only MST2 appears to be regulated at a protein level, we asked whether the Hippo kinases expression were regulated at a transcriptional level. To test this hypothesis, we evaluated whether decreased expression of the Hippo kinases at the protein level was due to decreased expression of the Hippo kinases at the RNA level. Quantitative RT-PCR was performed on all 12 sarcoma cell lines and expression normalized to GCT. Approximately 42% of cell lines demonstrated loss of expression of *MST1* at the RNA level, while 25% of cell lines demonstrated loss of expression of *MST2* at the RNA level (Figure 5A). In contrast, loss of expression of the *LATS1* and *LATS2* kinases at the RNA level was less frequently observed. Approximately 8% of sarcoma cell lines demonstrated loss of expression of *LATS1* at the RNA level, while no cell lines demonstrated loss of expression of *LATS2* at the RNA level.

### Expression of *MST1* and *MST2* are modestly regulated by promoter hypermethylation

The above findings suggest that loss of expression of *MST1* and *MST2* are regulated at a transcriptional level, while loss of expression of *LATS1* and *LATS2* are regulated by other mechanisms. Several authors have noted promoter hypermethylation of *MST1*, *MST2*, *LATS1*, and *LATS2* in various cancers [43–45]. However evaluation of how promoter methylation affects expression has not been comprehensively evaluated. To determine whether promoter hypermethylation affected expression of the Hippo kinases, we evaluated TCGA methylation data acquired from Firebrowse.org [48] (Supplementary Figure 6). A modest negative correlation was identified between methylation of CpG islands and RNA expression for *MST2* (*r* = −0.4) (Figure 5B).

To begin addressing whether hypermethylation of these promoters is functionally significant, we treated sarcoma cell lines with 10 μM 5-azacytidine (a covalent inhibitor of DNA methyltransferase I [49]) for 4 days. Quantitative RT-PCR demonstrated 6 cell lines that approximated at least a 1.5 fold increase in expression, however, only the RD cell line demonstrated a fold change increase greater than 2 in expression of *MST1*. Similarly, treatment with 5-azacytidine demonstrated 2 cell lines, RD and SW872, that had a greater than 2-fold increase in expression of *MST2*. Altogether, the data demonstrate that expression of *MST1* and *MST2* are modestly regulated by methylation of DNA, mirroring the TCGA *in silico* data. None of the cell lines demonstrated a two-fold increase in expression of *LATS1* or *LATS2* with treatment with 5-azacytidine (Figure 5C).

### Expression of MST1 and MST2 is silenced by histone deacetylation

Since treatment with 5-azacytidine (AZA) did not significantly rescue expression of *MST1* and *MST2* in many sarcoma cell lines, it suggested that promoter hypermethylation was not the predominant transcriptional mechanism regulating expression of *MST1* and *MST2*. Promoter hypermethylation is linked structurally to histone acetylation. Histone deacetylases are known to bind to methylated DNA via the MBD2/3 and MeCP2 adaptor proteins [50, 51].

**Figure 6:**
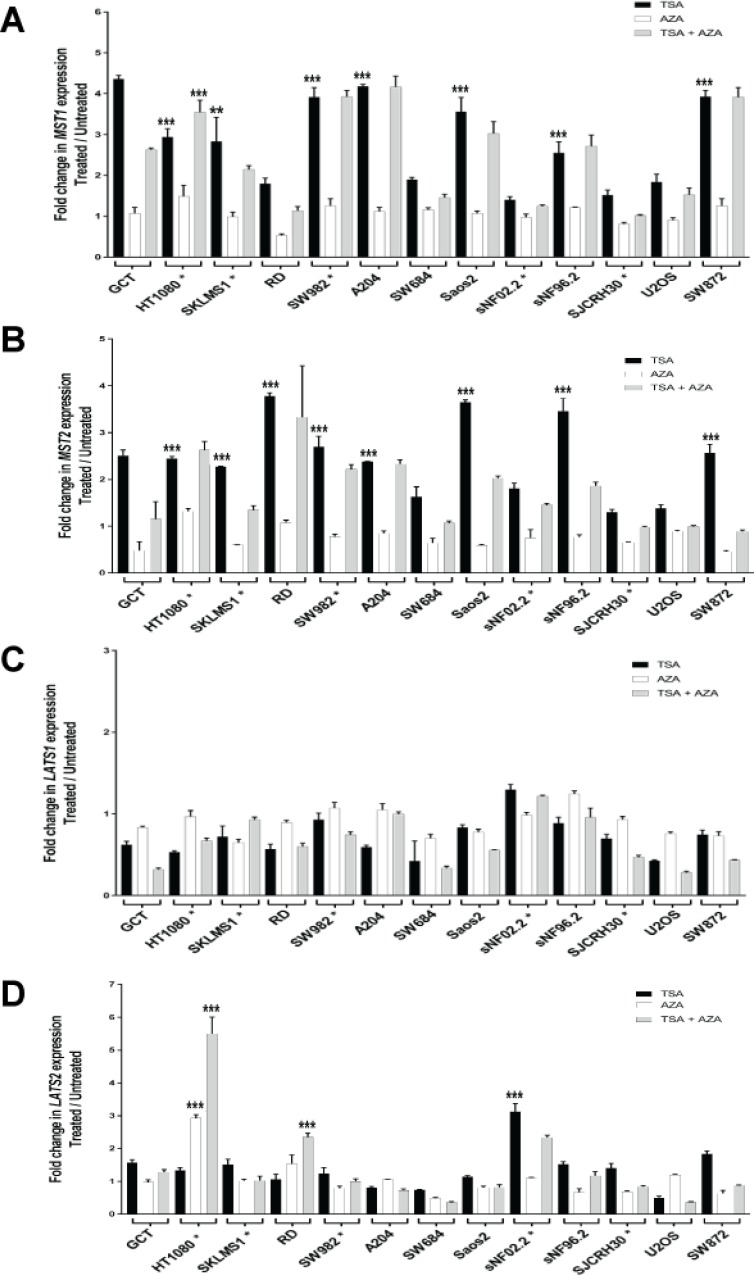
Increase in expression of the Hippo kinases after treatment with trichostatin-A (0.5 μM), 5-azacytidine (5 μM), and trichostatin-A (0.5 μM) plus 5-azacytidine (5 μM) unless indicated by asterisk SJCRH30^*^ cell line was treated with 0.125 μM trichostatin-A, SNF02.2^*^ was treated with 0.25 μM trichostatin-A. HT1080^*^, SKLMS1^*^, and SW982^*^ cell lines were treated with 1 μM 5-azacytidine. Increase in expression after treatment with trichostatin-A and/or 5-azacytidine is defined as a two-fold or greater increase in expression after treatment. (**A**) Trichostatin-A (TSA) stimulated an increase in expression of *MST1* in 7 of 12 sarcoma cell lines (58%). 5-azacytidine (AZA) at 5 μM did not cause an increase in expression of *MST1*. TSA and AZA had a statistically significant additive effect in the HT1080 cell line only. (**B**) Trichostatin-A (TSA) stimulated an increase in expression of *MST2* in 8 of 12 sarcoma cell lines (67%). At 5 μM 5-azacytidine, there is no increase in expression of *MST2*. No additive effect of adding 5-azacytidine and trichostatin-A was identified in the sarcoma cell lines. (**C**) No increase in expression in *LATS1* was identified with TSA, AZA, or TSA plus AZA. (**D**)TSA drove an increase in expression of *LATS2* in 1 of 12 sarcoma cell lines (8%). AZA caused an increase in expression of LATS2 in the HT1080 cell line. A statistically significant additive effect was seen in the HT1080 and RD cell lines with simultaneous treatment with trichostatin A and 5-azacytidine. Statistical analysis is shown for cell lines/conditions demonstrating a two-fold or greater decrease in expression with TSA or AZA treatment alone. Stastistical analysis is also shown for cell lines demonstrating an additive effect with treatment with TSA and AZA. Statistical significance determined by two-tailed *t*-test. ^*^ indicates *p* < 0.05; ^**^ indicates *p* < 0.01; ^***^ indicates *p* < 0.001.

This suggested that the promoter hypermethylation might coordinately regulate expression of the *MST1* and *MST2* kinases with histone deacetylation. To determine more directly whether promoter hypermethylation and histone deacetylation work in conjunction with one another to regulate expression of the Hippo kinases, we treated cells with 5 μM 5-azacytidine (AZA) for 4 days followed by treatment with 500 nM trichostatin A (inhibits class I, II, and IV histone deactylases (HDACs) by interacting with the Zn(II) in the catalytic site [52]) for 12 h (Figure 6A–6D). A lower concentration of 5-azacytidine was used in the majority of cell lines, rather than 10 μM as in previous experiments, so that addition of trichostatin A could be tolerated in the cell lines. Some lines could only tolerate lower levels of trichostatin A, including SJCRH30 (0.125 μM trichostatin A) and SNF02.2 (0.25 μM trichostatin A). The HT1080, SKLMS1, and SW982 cell lines could only tolerate 1 μM 5-azacytidine in combination with trichostatin A. Trichostatin A (TSA) and AZA treatment alone controls were included. Seven of the 12 sarcoma cell lines (58%) demonstrated a two-fold or greater increase in expression of *MST1* with treatment with TSA. TSA and AZA had a statistically significant additive effect in the HT1080 cell line only (Figure 6A). TSA stimulated an increase in expression of *MST2* in 8 of 12 sarcoma cell lines (67%) (Figure 6B). No increase in expression of *LATS1* was seen with TSA alone (Figure 6C). TSA promoted an increase of expression of *LATS2* in 1 of 12 sarcoma cell lines (8%). A statistically significant additive effect was seen with regards to *LATS2* expression in the HT1080 and RD cell lines with simultaneous treatment with trichostatin A (TSA) and 5-azacytidine (AZA) (Figure 6D).

The effectiveness of TSA in the above experiments in promoting histone acetylation was validated in three cell lines, RD, Saos2, and sNF96.2 that demonstrated an increase in expression of *MST1* and *MST2.* These three lines were treated with 0.5 μM TSA for 24 hours. Lysates were then probed with a pan-acetyl histone H3 antibody recognizing acetylated K9, K14, K18, K23, and K27 (Supplementary Figure 7A) confirming an increase in pan-acetylation of histone H3. To further validate the reversal in expression of *MST1* and *MST2* seen with treatment with TSA, we treated with a different HDAC inhibitor, N-(2-Aminophenyl)-4- [N-(pyridine-3ylmethoxycarbonyl)aminomethyl]benzamide (MS-275) in the same above three lines. Treatment with 1 μM MS-275 for 24 hours resulted in at least a 1.5 fold increase in expression of *MST1* and *MST2* in the three cell lines (Supplementary Figure 7B and 7C), confirming that reversal of expression with treatment with TSA was not an off-target effect but due to HDAC inhibition.

## DISCUSSION

Although TAZ and YAP have been acknowledged as central oncoproteins in multiple cancers including breast, colon, liver, lung, pancreatic, and thyroid cancers [33], the dominant paradigm is that these two oncoproteins are activated relatively late in tumor progression. This is due to the observation that few mutations of TAZ and YAP or the upstream Hippo kinases are activated. Challenging this paradigm has been the discovery of the TAZ-CAMTA1 [36–38] and YAP-TFE3 [39] fusion proteins in epithelioid hemangioendothelioma (EHE), a vascular sarcoma. EHE contains a t(1;3)(p36;q25) [36–38] or t(X;11) [39]chromosomal translocations encoding the TAZ-CAMTA1 and YAP-TFE3 fusion proteins, respectively. EHE contains few other cytogenetic alterations other than the above mentioned chromosomal translocations, indicating that these chromosomal translocations and gene fusions are the initiating event. The TAZ-CAMTA1 fusion protein [38] and presumably the YAP-TFE3 fusion protein function by constitutively activating the N terminal portion of TAZ and YAP so that they are no longer negatively regulated by the Hippo pathway. Expression of the TAZ-CAMTA1 fusion protein has been demonstrated to transform cells and promote various hallmarks of cancer [38], demonstrating that activated TAZ and YAP can serve as the initiating oncoproteins in cancers.

The question of whether TAZ and YAP serve as the initiating oncoproteins in other cancers is still largely unresolved. Because the preponderance of evidence indicates that the Hippo kinases are inactivated and that TAZ and YAP are activated by a complex interaction with multiple signal transduction pathways, the dominant view is that they must be activated later in tumor progression due to the time required for mutations to occur in other signal transduction pathways [53]. The above paradigm focuses on post-translational mechanisms by which the Hippo kinases, TAZ, and YAP can be regulated. In this study we identified several mechanisms via which the Hippo kinases can directly be down-regulated, suggesting that TAZ and YAP might be activated earlier in tumor initiation/progression than previously stipulated.

**Figure 7:**
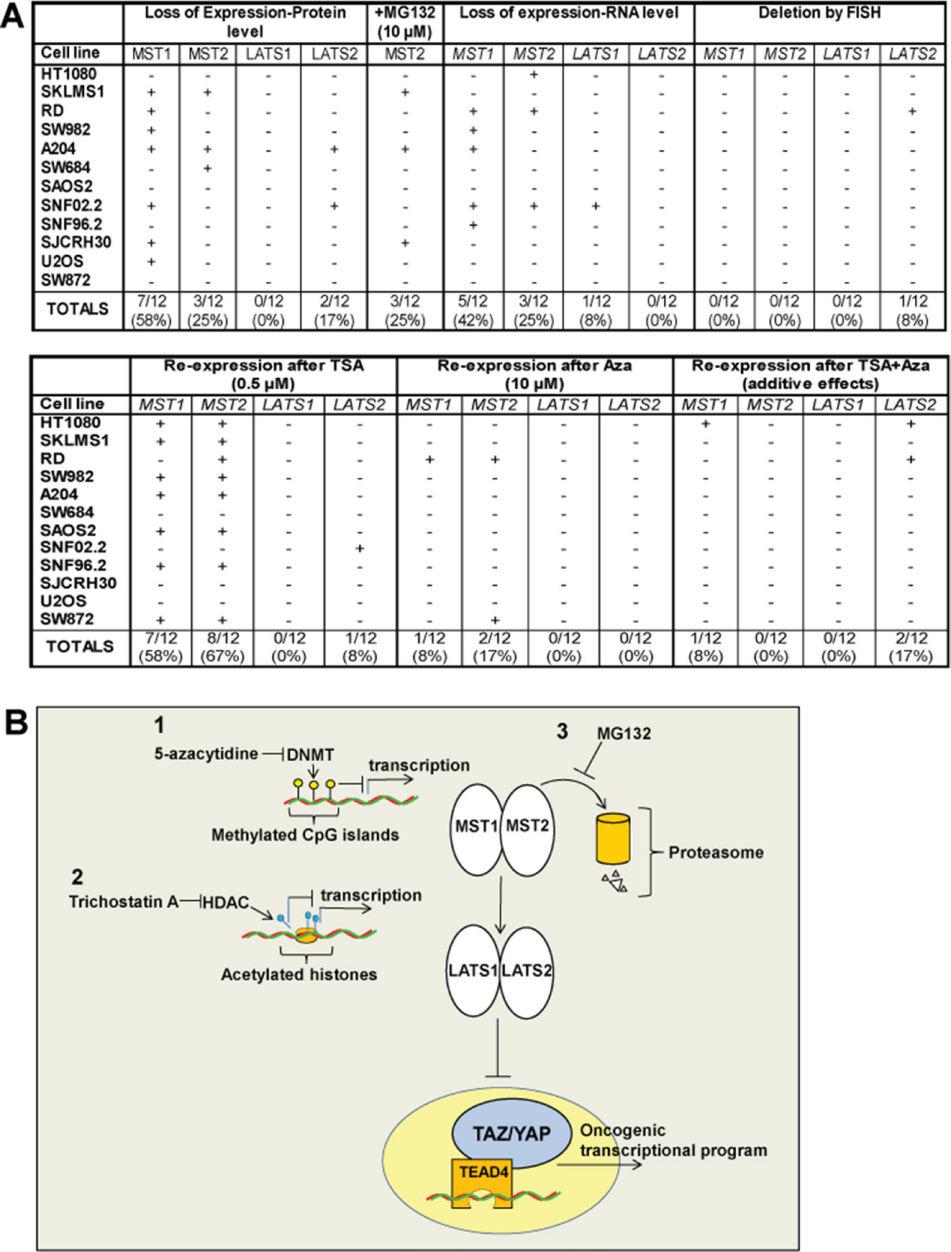
(**A**) Summary of cell lines diagram. Expression of the Hippo kinases was lost at the protein level in 0% (LATS1) to 58% (MST1) of the sarcoma cell lines, indicated by (+). Accumulation of the Hippo kinases with treatment with MG132, indicated by (+), was noted only for MST2, indicating that proteosomal degradation is an important mechanism by which MST2 expression is lost. Loss of expression at the RNA level was identified for *MST1* (42%) and *MST2* (25%) of sarcoma cell lines. Loss of expression at the RNA level for *LATS1* and *LATS2* was negligible. Deletions of the Hippo kinases were essentially absent from the sarcoma cell lines with the exception of *LATS2*, where 1 of the 12 sarcoma cell lines (8%) demonstrated a deletion. Treatment with 10 μM 5-azacytidine resulted in a modest increase in expression in 8–17% of the sarcoma cell lines. Treatment with 0.5 μM TSA resulted in a reversal of expression in a higher percentage of cell lines, predominantly with *MST1* and *MST2*. Treatment with TSA and 5-azacytidine showed an additive effect with regards to re-expressing the Hippo kinases in some cell lines. (**B**) Expression of the Hippo kinases is regulated by at least three different mechanisms shown in this model, potentially targetable by different therapeutic interventions. Promoter (CpG island) hypermethylation is one mechanism that appears to modestly regulate the expression of predominantly *MST1* and *MST2*. Histone deacetylation can also promote silencing the expression of the Hippo kinases, again particularly *MST1* and *MST2*, and to a lesser degree *LATS2*. Proteosomal degradation plays a role in in regulating the expression of MST2 and could be targeted as well.

Herein, utilizing an unbiased approach with clinical samples, we showed in sarcomas that the Hippo kinases can be primarily dysregulated by a variety of mechanisms resulting in their loss of expression (Figure 7A, 7B). Loss of expression of the Hippo kinases was identified in clinical sarcoma samples demonstrating activated TAZ and YAP at a relatively high level, ranging from 19% (LATS1) to 47% (MST1). The majority of TAZ/YAP activated clinical sarcoma samples (75%) demonstrated loss of expression of one of the Hippo kinases, while other combinations of loss of expression of the Hippo kinases was much less frequent, suggesting that loss of expression of one of the Hippo kinases may be sufficient to activate TAZ or YAP. These findings were mirrored in sarcoma cell lines that demonstrated constitutive activation of TAZ/YAP which demonstrated a similar frequency with regards to loss of expression of at least one of the Hippo kinases (83%) (Figure 7A).

Identifying sarcoma cell lines that demonstrated loss of expression of the Hippo kinases allowed us to identify various mechanisms by which this loss of expression was occurring. These mechanisms include: regulation by ubiquitin-mediated degradation, as well as two distinct methods of epigenetic regulation, promoter hypermethylation and histone deacetylation (Figure 7B). The Itch E3 ubiquitin protein ligase has already been implicated in regulation of LATS1 [41, 42], we also identify that MST2's expression is negatively regulated by a ubiquitin ligase in several sarcoma cell lines. Additional studies to identify this ubiquitin ligase is needed. The finding that LATS2 expression decreased with MG132 treatment in some sarcoma cell lines suggests that treatment with proteosomal inhibitors for sarcomas with decreased MST2 expression may not be effective. Additional pre-clinical studies to study these potentially competing effects are warranted.

The presence of promoter hypermethylation in the Hippo kinases, including all four of the Hippo kinases has been noted for some time in sarcomas, astrocytomas, and breast cancer [43–45]. Although some studies showed a correlation between mRNA levels and the degree of promoter hypermethylation, it was unclear how tightly promoter hypermethylation regulated Hippo kinase expression. Our *in silico* analysis and *in vitro* experiments demonstrate that promoter hypermethylation modestly regulates Hippo kinase expression, but that it can be reversed in some contexts with DNA methyltransferase inhibitors such as 5-azacytidine. Because the effects of treatment with 5-azacytidine was modest, it suggested that other epigenetic mechanisms may be responsible for regulating expression of the Hippo kinases. Since promoter hypermethylation and histone deacetylation are tightly linked processes, due to the presence of adaptor proteins that are present, we hypothesized that histone deacetylation may play a role in negatively regulating the expression of the Hippo kinases. Indeed, inhibition of histone deactylase by trichostatin A resulted in a greater than 2 fold increase in *MST1* and *MST2* expression in the majority of cell lines, confirming that histone deacetylation plays a role in the regulation of *MST1* and *MST2*. This data suggests a new mechanism of dampening *MST1* and *MST2* expression by histone deacetylation.

Additional preclinical studies are required to evaluate the feasibility of upregulating expression of the Hippo kinases in patients with TAZ/YAP activated sarcomas by modulating proteosomal degradation of MST2, and potentially inhibiting DNA methyltransferases and histone deacetylases.

## MATERIALS AND METHODS

### Bioinformatics analysis

The Cancer Genome Atlas (TCGA) methylation data (acquired from Firebrowse.org) [48], utilized the following methylation array platforms: Illumina Infinium HumanMethylation27, Illumina Infinium HumanMethylation450, Illumina DNA Methylation OMA002, Illumina DNA Methylation OMA003, and the following gene expression platforms: Agilent 244K Gene Expression G4502A-07-1, Agilent 244K Gene Expression G4502A-07-2, Agilent 244K Gene Expression G4502A-07-3, Affymetrix Human Exon 1.0 ST Array, Affymetrix HT Human Genome U133 Array. Analysis included 259 sarcomas.

### Tissue microarray construction

A total of 159 untreated sarcomas were retrieved from the University of Iowa Department of Pathology with previous approval from the Institutional Review Board. The tissue microarray was constructed by arraying 1.0 mm cores taken from formalin fixed paraffin embedded tissue and assembled using a MTA-1 tissue arrayer from Beecher Instruments (Sun Prairie, WI) as previously described [35]. Sarcomas were classified according to World Health Organization criteria [54].

### Antibodies

Anti-TAZ (mouse monoclonal 1H9; catalog # LS-C173295) utilized for immunohistochemistry (1:50) and immunofluorescence (1:100) was obtained from LifeSpan BioSciences (Seattle, WA, USA). Anti-YAP (rabbit polyclonal, catalog #sc-15407) utilized for immunohistochemistry (1:100) and immunofluorescence (1:100) was obtained from Santa Cruz Biotechnology (Santa Cruz, CA, USA). Anti-TAZ (rabbit polyclonal, catalog# HPA007415) utilized for Western blot was obtained from Sigma-Aldrich (St. Louis, MO, USA). Anti-YAP (D8H1X XP; catalog #14074) utilized for Western blot was obtained from Cell Signaling (Danvers, MA, USA). A pan-acetyl histone H3 antibody (rabbit polyclonal, catalog# ab47915) was obtained from Abcam (Cambridge, MA, USA).

Anti-MST1 (rabbit monoclonal [EPR6207], catalog# ab124787) utilized for immunohistochemistry (1:200) and western blot (1:1000) was obtained from Abcam. Anti-MST2 (rabbit monoclonal [EP1466Y], catalog# ab52641) utilized for western blot (1:1000) was obtained from Abcam (Cambridge, MA, USA). Anti-MST2 (rabbit polyclonal, catalog# PA5-17691) utilized for immunohistochemistry (1:2000) was obtained from ThermoFisher Scientific (Waltham, MA, USA). Anti-LATS1 (goat polyclonal, catalog# sc-12494) utilized for immunohistochemistry (1:100) and western blot (1:200) was obtained from Santa Cruz (Santa Cruz, CA, USA). Anti-LATS2 (rabbit polyclonal, catalog# 20276-1-AP) utilized for western blot (1:500) was obtained from ProteinTech (Rosemont, IL, USA). Anti-LATS2 (rabbit polyclonal, catalog# ab70565) utilized for immunohistochemistry (1:100) was obtained from Abcam (Cambridge, MA, USA). Antigen retrieval using TRIS buffer (pH 9) was done on immunohistochemistry samples.

Anti-β-actin (AC-15; catalog #A5441) was obtained from Sigma-Aldrich (St. Louis, MO, USA). Alexa 568 conjugated secondary antibody (catalog# A11031 or A11036) was obtained from Invitrogen-Life Technologies (Grand Island, NY, USA). Horseradish peroxidase-conjugated secondary antibodies used for western blots were obtained from Santa Cruz Biotechnology (catalog# sc-2055, sc-2054, or sc-2033).

### Western blot

Harvested cells were lysed in radioimmunoprecipitation assay (RIPA) complete lysis buffer with the addition of Complete Protease Inhibitor Cocktail (Roche) and PhosSTOP Phosphatase Inhibitor Cocktail (Roche) according to the manufacturer's instructions. Total protein concentration was measured using Pierce BCA™ Protein Assay Kit (ThermoFisher Scientific, Waltham, MA). Proteins were transferred to a polyvinylidene difluoride (PVDF) membrane and probed with antibodies described above. Immediately after transfer, membranes were stained with 10 mLs of Ponceau S (0.1% (w/v) in 5% acetic acid) (Sigma-Aldrich, catalog# P7170) [55].

### Quantitation of Western blots

Samples utilized for quantiative Western blot were run in triplicate. Western blot images were exported as tiff files and converted to grey scale jpeg images with Photoshop. The images were then quantitated in Image J using “Grey Mean Value” measured using a previous described protocol [56]. A region of interest was defined and used across blots. Protein of interest and Ponceau S loading control values were acquired, densities were inverted and background values were subtracted. Net protein/net loading control ratios were calculated for each protein. The ratios were then normalized to an internal control (cell line or cell line/treatment condition) on each blot.

### Immunofluorescence staining

Cell lines were fixed with 4% paraformaldehyde (in 1X PBS) for 15 min. After washing with PBS, cells were permeabilized and blocked with 0.3% Triton X-100 and 3% fetal bovine serum for 30 min. Cells were incubated with anti-TAZ and anti-YAP antibody diluted (1:100) in 3% fetal bovine serum at 4°C overnight in a humidity chamber. The primary antibody was removed, cells washed, then incubated with Alexa Fluor 488-conjugated secondary antibody (Invitrogen-Life Technologies) for 45 minutes to 1 hr at room temperature. Immunofluorescence was visualized using a Nikon Eclipse E600 microscope (Tokyo, Japan) with SPOT imaging software or an Olympus BX-61 microscope (Tokyo, Japan) with cellSens imaging software.

### Cell culture

Sarcoma cell lines were obtained from the American Type Culture Collection (ATCC, Manassas, VA, USA) and were cultured in DMEM, RPMI, or McCoy's media (according to ATCC recommendations) containing 10% Fetal Bovine Serum (Invitrogen-Life Technologies), 1 mM sodium pyruvate, and 50μg/mL pen/strep. All cells were cultured at 37°C and 5% CO_2_. To detect proteosomal degradation, 10 μM MG132 was added to cells for 12 hours. To detect reconstitution of the Hippo kinases, either 5 or 10 μM 5-azacytidine (Sigma-Aldrich) was added to cell lines for 4 days. When 5-azacytidine and trichostatin A (Sigma-Aldrich) were combined, 0.125 μM, 0.25 μM, or 0.5 μM trichostatin A (maximal amount tolerated in combination with 5-azacytidine) was added for 12 hours on day 4 of the 5-azacytidine treatment. 1 μM MS-275 (Tocris, Minneapolis, MN), an additional HDAC inhibitor, was added to cell lines for 24 hours.

### Fluorescence *in situ* hybridization

Bacterial artificial chromosome (BAC) clones that hybridized to *MST1*(*STK4*) (20q13.12), *MST2*(*STK3*) (8q22.2), *LATS1* (6q25.1), and *LATS2* (13q12.11) were obtained from Children's Hospital Oakland Research Institute (Oakland, CA). For *MST1*(*STK4*) the BAC utilized was RP11-844G5 (195 kb), for *MST2*(*STK3*) the BACs utilized were RP11-208K6 (167 kb), RP11-825F16 (187 kb), and RP11-159A16 (213 kb), for *LATS1* the BAC utilized was RP11-69I17 (159 kb), for *LATS2* the BAC utilized was RP11-45A5 (154 kb) and RP11-22J15 (202 kb). BACs were isolated utilizing the QIAGEN Large-Construct Kit (Qiagen, Germantown, MD). 1 μg of BAC DNA was nick translated utilizing the Nick Translation Kit (Abbott Molecular Inc., Des Plaines, IL) and Spectrum Orange dUTP (Abbott Molecular Inc.). Unincorporated fluorescent labeled nucleotides were cleaned up using NucAway Spin Columns (Invitrogen by ThermoScientific, Carlsbad, CA), according to the manufacturer's instructions. An ethanol precipitation of the eluant was performed, and probes were resuspended in 10μL molecular grade H_2_O. FISH studies were carried out using the above mentioned probes. Hybridizations were performed on ThermoBrite at a melt temperature of 75°C for 2 minutes. After overnight hybridization at 37°C, the slides were washed in 0.4XSSC/0.3% NP-40 for 2 minutes at 76°C and in 2XSSC/0.1% NP-40 for 1 minute at room temperature. The slides were then counterstained with DAPI. Slides were analyzed and images acquired through the CytoVision computerized imaging system (Leica, USA). 100 interphase nuclei were counted for each probe. Metaphase analysis was performed to confirm hybridization of FISH probes to the appropriate chromosome.

### Quantitative reverse transcription polymerase chain reaction

Total RNA was isolated from sarcoma cell lines using TRIzol reagent (Invitrogen-Life Technologies). Total RNA was treated with DNase (Invitrogen-Life Technologies), and then column purified using the PureLink RNA mini kit (Ambion-Thermo Fisher Scientific). 1 μg of DNase treated RNA was converted to cDNA using Superscript III Reverse Transcriptase (Invitrogen-Life Technologies) and 50 ng of random primers (Promega, Madison, WI USA). PCR amplification was performed in technical triplicates on the Applied Biosystems QuantStudio 3 Real-Time PCR System (Applied Biosystems-Life Technologies). Relative quantitation was performed utilizing the delta-delta C_T_ method and the geometric mean of *ACTB* (β-actin) and *POLR2A* (RNA Polymerase II) C_T_ values as the reference control. The TaqMan Universal PCR Master Mix (Applied Biosystems-Life Technologies) was utilized as well as PrimeTime standard qPCR primer/probe sets from Integrated DNA Technologies (Iowa City, IA, USA). The Taqman based approach utilized the following primers and probes:

*MST1(STK4)-F 5′-TTGACACTCCTTTGGCACTC-3′*; *MST1(STK4)-R 5′-CCTCCCACATTCCGAAAACC-3′*; *MST1(STK4) Probe 5′-CAGCTCCTGCAGCACCCAT TTG-3′*; *MST2(STK3)-F 5′-GATTTAAGAATGGTTGCAAT TTCATCT-3′ MST2(STK3)-R 5′-GACCTCTGGATTGTTA TGGAG T-3′*; *MST2*(*STK3*) Probe 5′-TGGCGCTGGCTC TGTCTCA-3′;*LATS1* -F 5′-GTGAAGAGATGTTTGC CAGTTG-3′; *LATS1*-R 5′-AGTTGTGTGATTGGTGGA GTG-3′; *LATS1* Probe 5′-TGTTTGTGCCAAGAAAGGAGGTTGTC-3′; *LATS2*-F 5′-ACACCGACAGTTAGACA CATC-3′; *LATS2*-R 5′-AACTCACAGATTTCGGCCTC-3′; *LATS2* Probe 5′-ACAGGACAGCATGGAGCCCAG-3′; *ACTB*-F 5′-ACCTTCTACAATGAGCTGCG-3′; *ACTB-*R 5′-CCTGGATAGCAACGTACATGG-3′; *ACTB* Probe 5′-ATCTGGGTCATCTTCTCGCGGTTG-3′; *POLR2A*-F 5′-TCAGCATGTTGGACTCGATG-3′; *POLR2A*-R 5′-CGTATTCGCATCATGAACAGC-3′; *POLR2A*- Probe 5′-ACCACCTCTTCCTCCTCTTGCATCT-3′; The following qPCR cycling conditions were used: 95°C^10:00^(95°C^0:15^, 60°C^1:00^)_40_.

### PCR amplification and sequencing of *WWTR1*(TAZ) and *YAP1*

Genomic DNA was isolated from sarcoma cell lines utilizing the DNeasy Blood and Tissue Kits (Qiagen) according to the manufacturer′s instructions. 20 ng of genomic DNA was amplified utilizing GoTaq DNA polymerase (Promega, Madison, WI) and the following PCR cycling conditions: 95°C^2:00^(95°C^0:30^, 63°C^0:30^, 72°C^0:30)^_35_ 72°C^10:00^. The following PCR primers were utilized:

*WWTR1* Exon 2-F 5′-GCCTAGCTCGTGGCGGAAGAAGATCCTGC-3′; *WWTR1* Exon 2-R 5′-GCAGTGGCAGCTCGTCGGTCACG-3′; *WWTR1* Exon 6-F 5′-GCAGCATGGCACAACTGCACTAG-3′; *WWTR1* Exon 6-R 5′-CTACCTGTATCCATCTCATCCA CATTGCTG-3′; *YAP1* Exon 1-F 5′-CGCCGGGCATCA GATCGTGC-3′; *YAP1* Exon 1-R 5′-GGACGACTCCA GTTCCACTTCGC-3′; *YAP1* Exon 2-F 5′-GCACCCA TAACTGCACTGACCTC-3′; *YAP1* Exon 2-R 5′-GTCTTTGCCATCTCCCAACCTGC-3′; *YAP1* Exon 7-F 5′-GTCTCTGTGCCACCACCACCTGGAG-3′; *YAP1* Exon 7-R 5′-CTGTATCCATCTCATCCACACTGTTCAGG-3′.

### Statistics

Standard deviation for the quantitative western blots was calculated from fold change expression derived from different western blot experiments. For quantitative RT-PCR, standard deviation was calculated from fold change values from each triplicate. Correlation between fraction methylation of CpG islands and expression of the Hippo kinases (RSEM [log_2_]) was calculated by Spearman's correlation coefficient.

## SUPPLEMENTARY MATERIALS FIGURES AND TABLES



## Abbreviations

TAZ: transcriptional coactivator with PDZ-binding motif
YAP: yes associated protein
AZA: 5-azacytidine
TSA: Trichostatin A
