# Supplementary material for: A comprehensive evaluation of Hippo pathway silencing in sarcomas
Source: Oncotarget. 2018 Aug 3;9(60):31620–36. doi: 10.18632/oncotarget.25824 (PMC6114978; doi:10.18632/oncotarget.25824)
Supplement: Supplementary file 1 [file oncotarget-09-31620-s001.pdf]

A comprehensive evaluation of Hippo pathway silencing in sarcomas

SUPPLEMENTARY MATERIALS

A

	MST1(STK4)	*MST2(STK3)*	*LATS1*	*LATS2*
% mutation	0.4%	0%	0%	0.8%

B

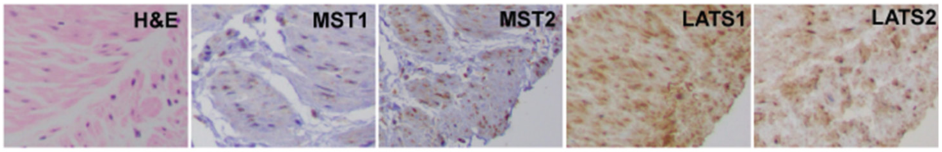

C

Sarcomas-histological type	MST1		MST2		LATS1		LATS2	
Alveolar/Sclerosing rhabdomyosarcoma	6/11	(55%)	9/11	(82%)	2/11	(18%)	2/11	(18%)
Synovial sarcoma	11/11	(100%)	6/11	(54%)	0/11	(0%)	4/11	(36%)
Malignant peripheral nerve sheath tumor	8/10	(80%)	5/10	(50%)	3/10	(30%)	3/10	(30%)
High grade osteosarcoma	4/9	(44%)	0/9	(0%)	8/9	(89%)	4/9	(44%)
Uterine leiomyosarcoma	3/7	(43%)	1/7	(14%)	6/7	(86%)	2/7	(29%)
Soft tissue leiomyosarcoma	5/10	(50%)	1/10	(10%)	3/9	(33%)	7/10	(70%)
Myxofibrosarcoma	4/8	(50%)	2/8	(25%)	0/7	(0%)	3/8	(38%)
Undifferentiated pleomorphic sarcoma	10/28	(36%)	2/28	(7%)	4/28	(14%)	6/28	(21%)
Clear cell sarcoma	2/4	(50%)	3/4	(75%)	1/4	(25%)	0/4	(0%)
Well-differentiated liposarcoma	4/7	(57%)	4/7	(57%)	5/9	(56%)	0/9	(0%)
Dedifferentiated liposarcoma	6/8	(75%)	3/8	(38%)	0/9	(0%)	2/9	(22%)
Myxoid/round cell liposarcoma	4/9	(44%)	5/9	(56%)	0/9	(0%)	2/9	(22%)
Epithelioid sarcoma	0/3	(0%)	0/3	(0%)	0/3	(0%)	2/4	(50%)
Angiosarcoma	0/4	(0%)	0/5	(0%)	0/4	(0%)	0/5	(0%)
Ewing's sarcoma	0/1	(0%)	1/1	(100%)	0/1	(0%)	0/1	(0%)
Pleomorphic liposarcoma	2/7	(29%)	0/7	(0%)	0/7	(0%)	4/7	(57%)
Chondrosarcoma	4/11	(36%)	4/10	(40%)	2/10	(20%)	4/11	(36%)
**Total**	**73/148**	**(49%)**	**46/148**	**(31%)**	**34/148**	**(23%)**	**45/153**	**(29%)**

D

Sarcoma-histological type	YAP or TAZ (+) and MST1/MST2 (-)		YAP or TAZ (+) and LATS1/LATS2 (-)		YAP or TAZ (+) and MST1/MST2, LATS1/LATS2 (-)	
Alveolar/Sclerosing rhabdomyosarcoma	4/9	(44%)	1/9	(11%)	1/9	(11%)
Synovial sarcoma	6/11	(55%)	0/11	(0%)	0/11	(0%)
Malignant peripheral nerve sheath tumor	3/9	(33%)	1/9	(11%)	0/9	(0%)
High grade osteosarcoma	0/6	(0%)	1/6	(17%)	0/6	(0%)
Uterine leiomyosarcoma	0/7	(0%)	2/7	(29%)	0/7	(0%)
Soft tissue leiomyosarcoma	1/8	(13%)	1/8	(13%)	0/8	(0%)
Myxofibrosarcoma	0/7	(0%)	0/7	(0%)	0/7	(0%)
Undifferentiated pleomorphic sarcoma	2/26	(8%)	3/26	(12%)	0/26	(0%)
Well-differentiated liposarcoma	0/1	(0%)	0/2	(0%)	0/1	(0%)
Dedifferentiated liposarcoma	1/8	(13%)	0/8	(0%)	0/8	(0%)
Myxoid/round cell liposarcoma	0/6	(0%)	0/6	(0%)	0/6	(0%)
Epithelioid sarcoma	0/3	(0%)	0/2	(0%)	0/2	(0%)
Angiosarcoma	0/4	(0%)	0/4	(0%)	0/3	(0%)
Pleomorphic liposarcoma	0/6	(0%)	0/6	(0%)	0/6	(0%)
Chondrosarcoma	0/3	(0%)	0/2	(0%)	0/1	(0%)
**Total**	**17/114**	**(15%)**	**9/113**	**(8%)**	**1/110**	**(1%)**

Supplementary Figure 1: (**A**) Mutations in the Hippo kinases are rare, ranging from 0% (*MST2* and *LATS1*) to 0.8% (*LATS2*). (**B**) Smooth muscle positive control (including H&E section) for MST1, MST2, LATS1, and LATS2. (**C**) Loss of expression of the Hippo kinases regardless of TAZ or YAP activation status. MST1 expression is lost in 49% of sarcomas. MST2 expression is lost in 31% of sarcomas. LATS1 expression is lost in 24% of sarcomas. LATS2 expression is lost in 29% of sarcomas. (**D**) Various combinations of loss of expression were evaluated. MST1 and MST2 expression together were lost in 15% of sarcomas, while LATS1 and LATS2 expression together were lost in 8% of sarcomas. Loss of expression of all four Hippo kinases was rare; only 1 of 110 sarcomas demonstrated this pattern of loss of expression.

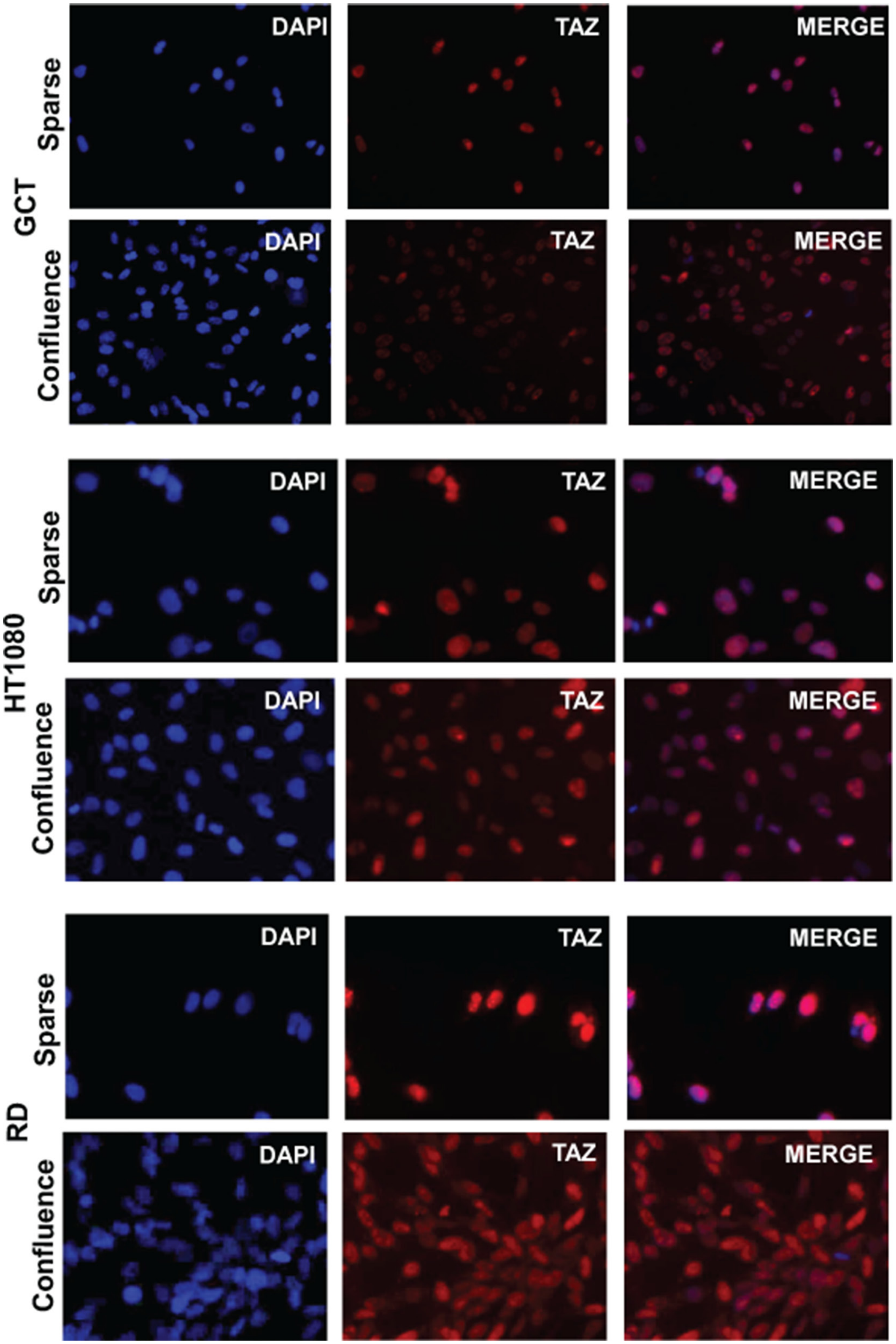

GCT
Sparse
Confluence
HT1080
Sparse
Confluence
RD
Sparse
Confluence
DAPI
TAZ
MERGE

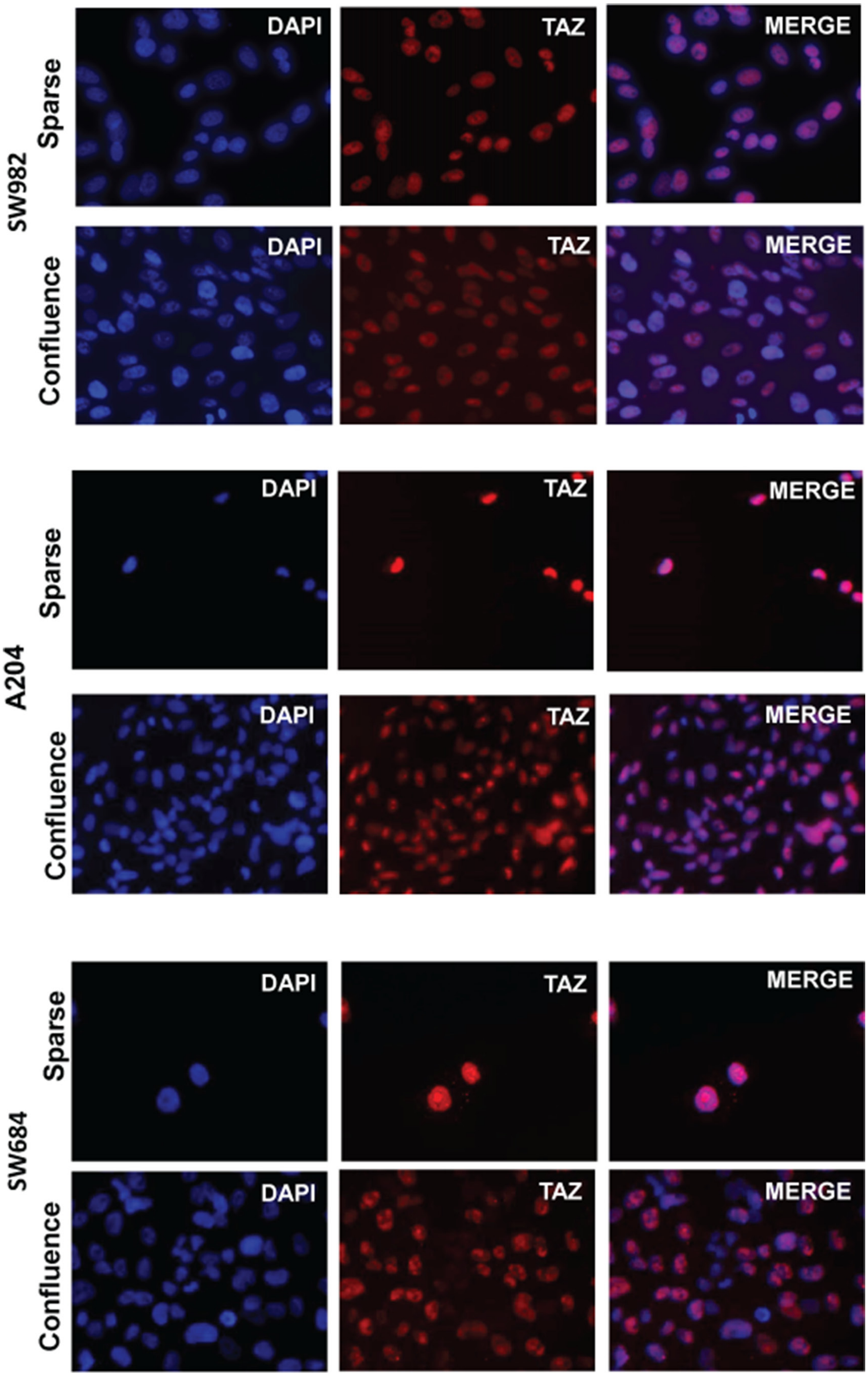
SW982
Sparse
Confluence
DAPI
TAZ
MERGE
A204
SW684

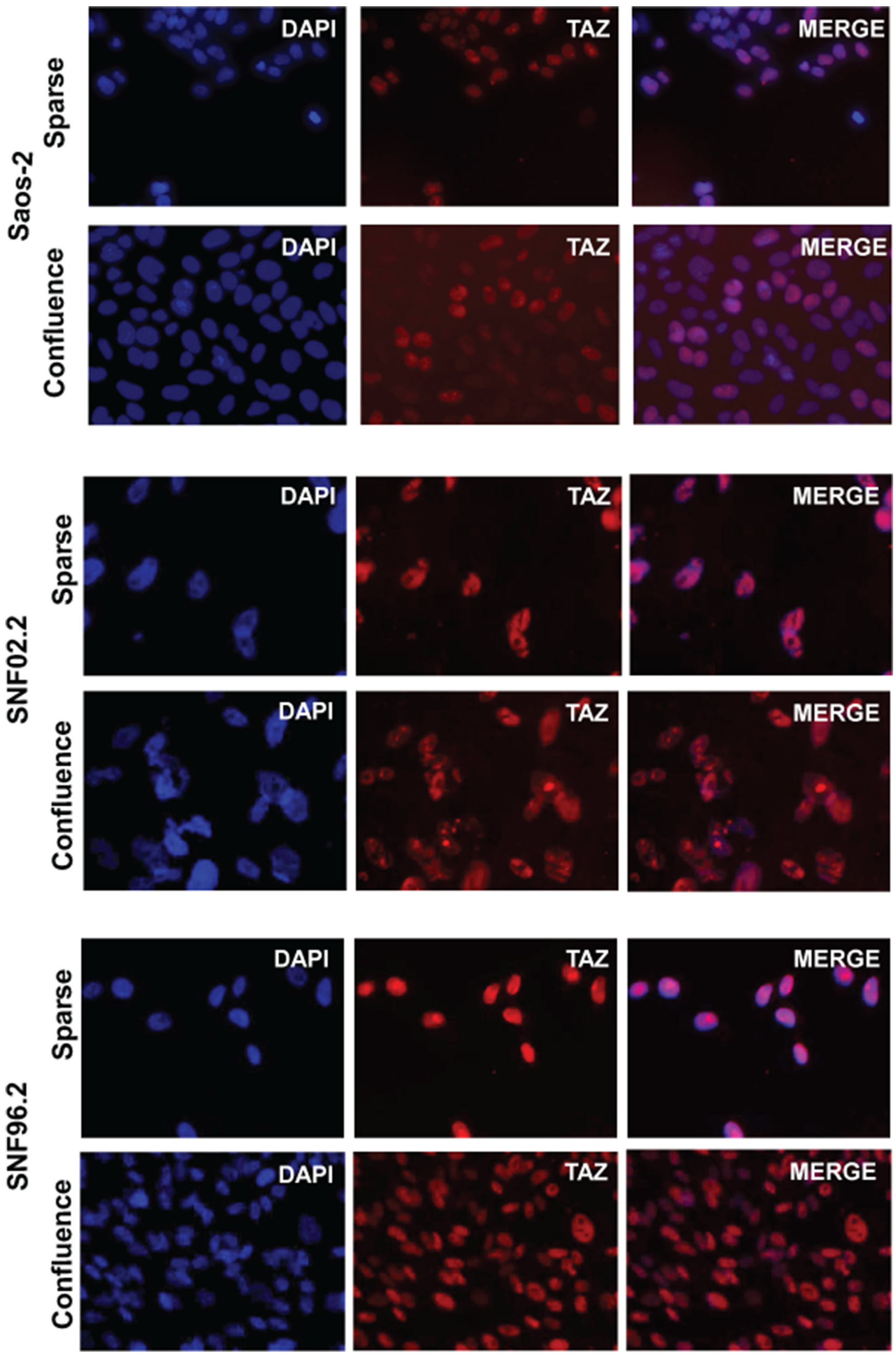

Saos-2
Sparse
Confluence
SNF02.2
Sparse
Confluence
SNF96.2
Sparse
Confluence
DAPI
TAZ
MERGE

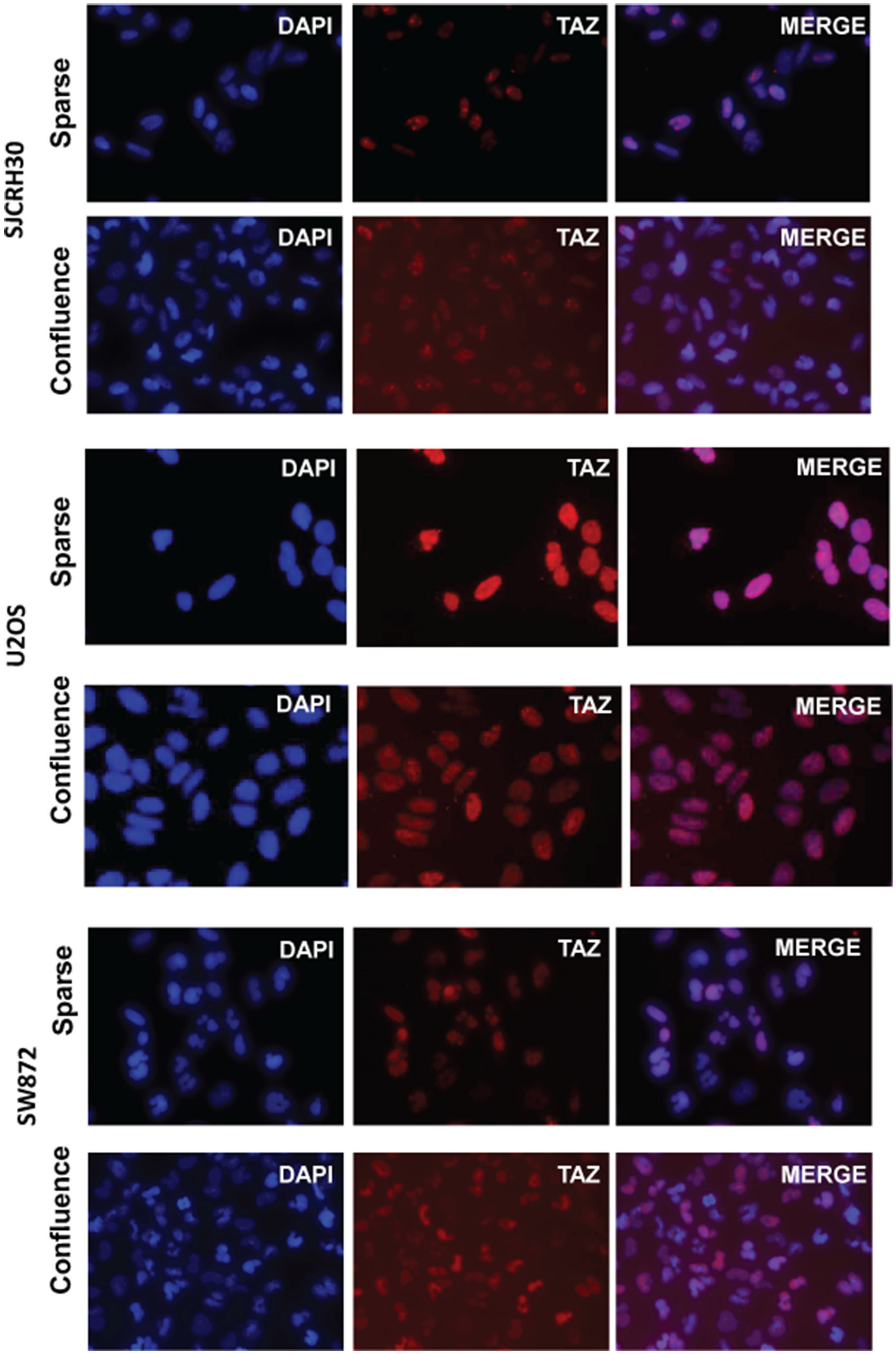

Supplementary Figure 2: Immunofluorescence for TAZ during sparse and confluent conditions. GCT demonstrates reduced nuclear localization of TAZ, indicating the Hippo pathway is still active in this cell line. The remaining sarcoma cell lines (HT1080, RD, SW982, A204, SW684, Saos2, SNF02.2, SNF96.2, SJCRH30, U2OS, and SW872) demonstrate TAZ within the nucleus.

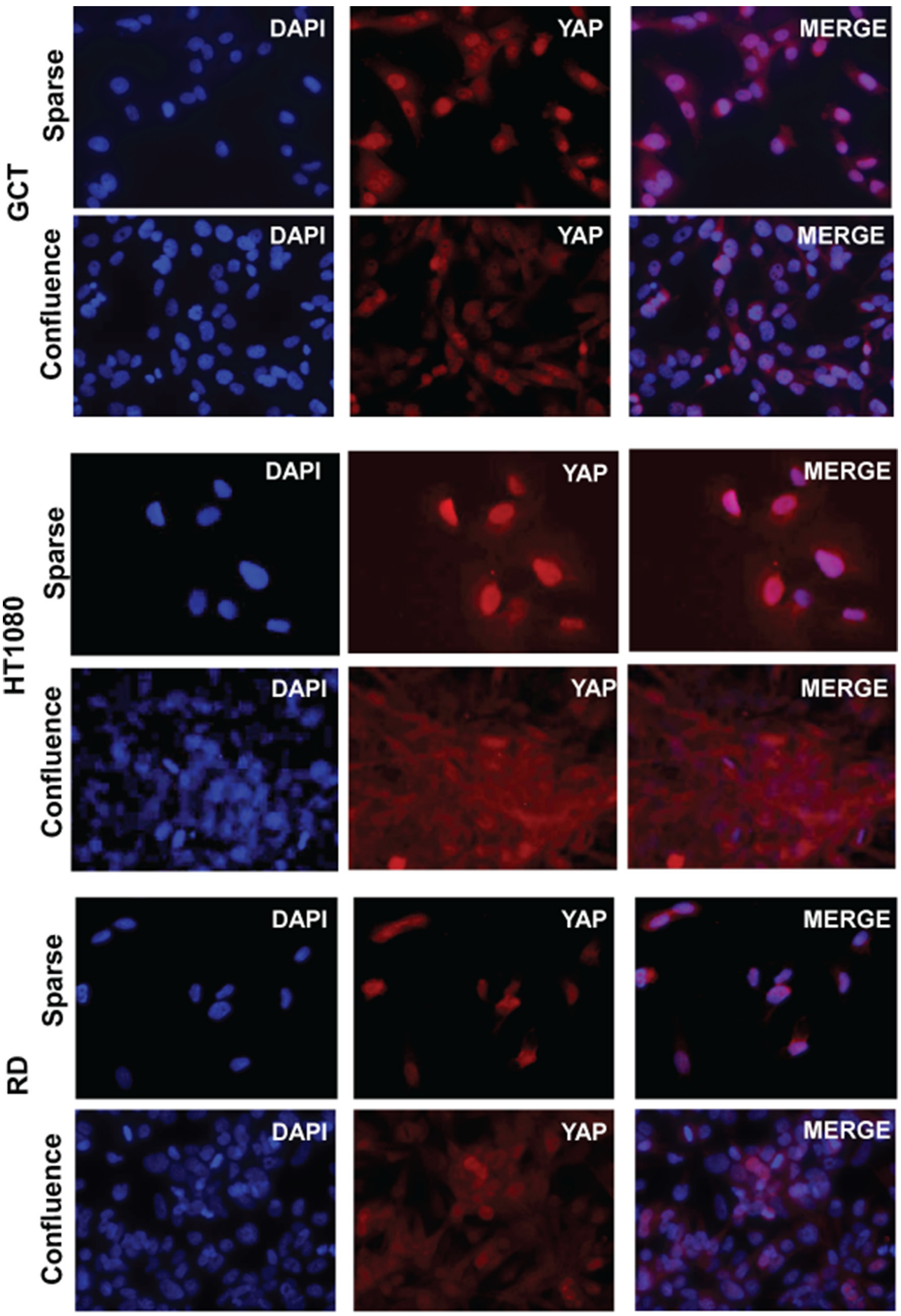
GCT
Sparse
Confluence
DAPI
YAP
MERGE
HT1080
Sparse
Confluence
DAPI
YAP
MERGE
RD
Sparse
Confluence
DAPI
YAP
MERGE

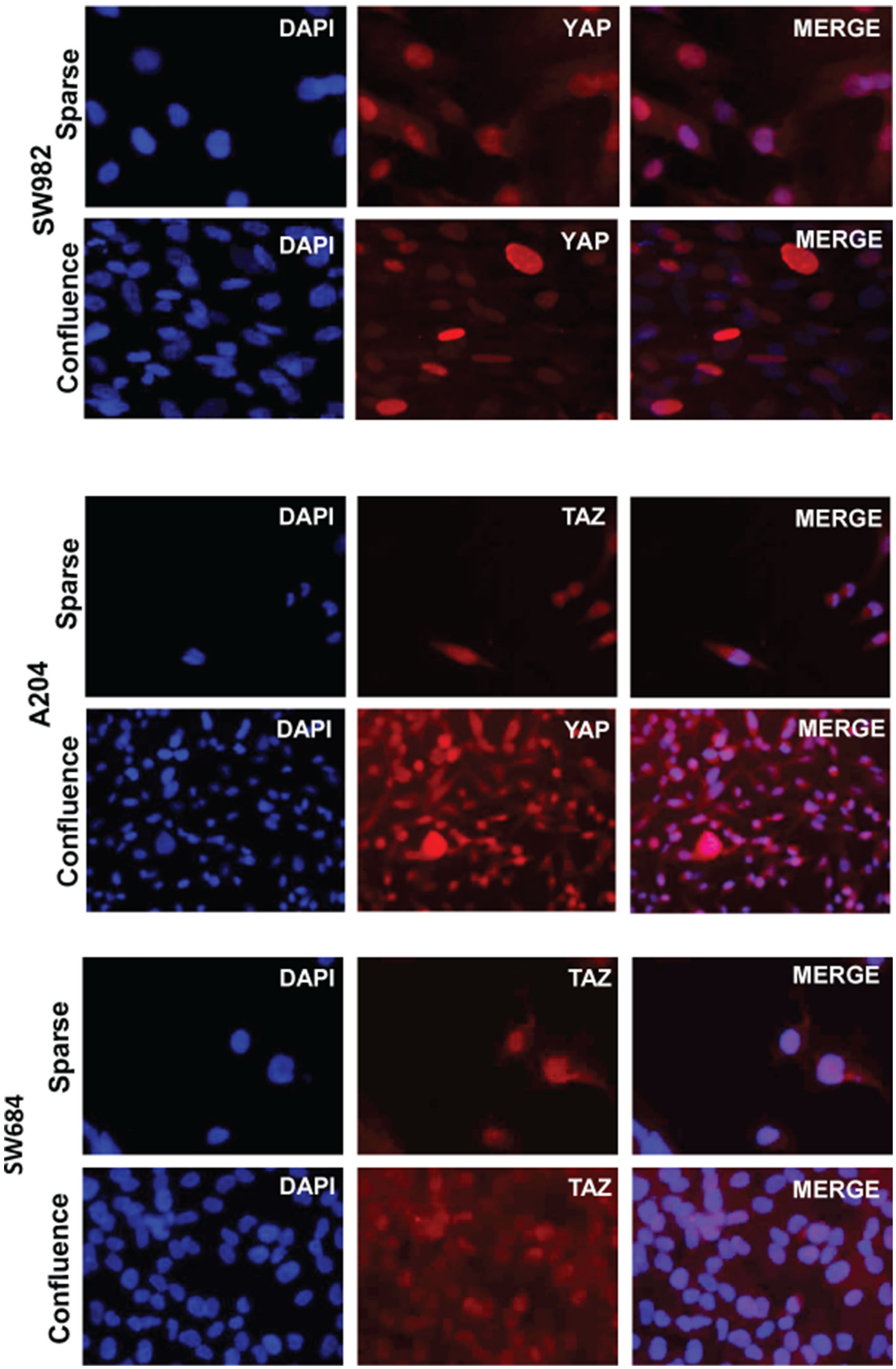
SW982
Sparse
Confluence
DAPI
YAP
MERGE
A204
TAZ
SW684

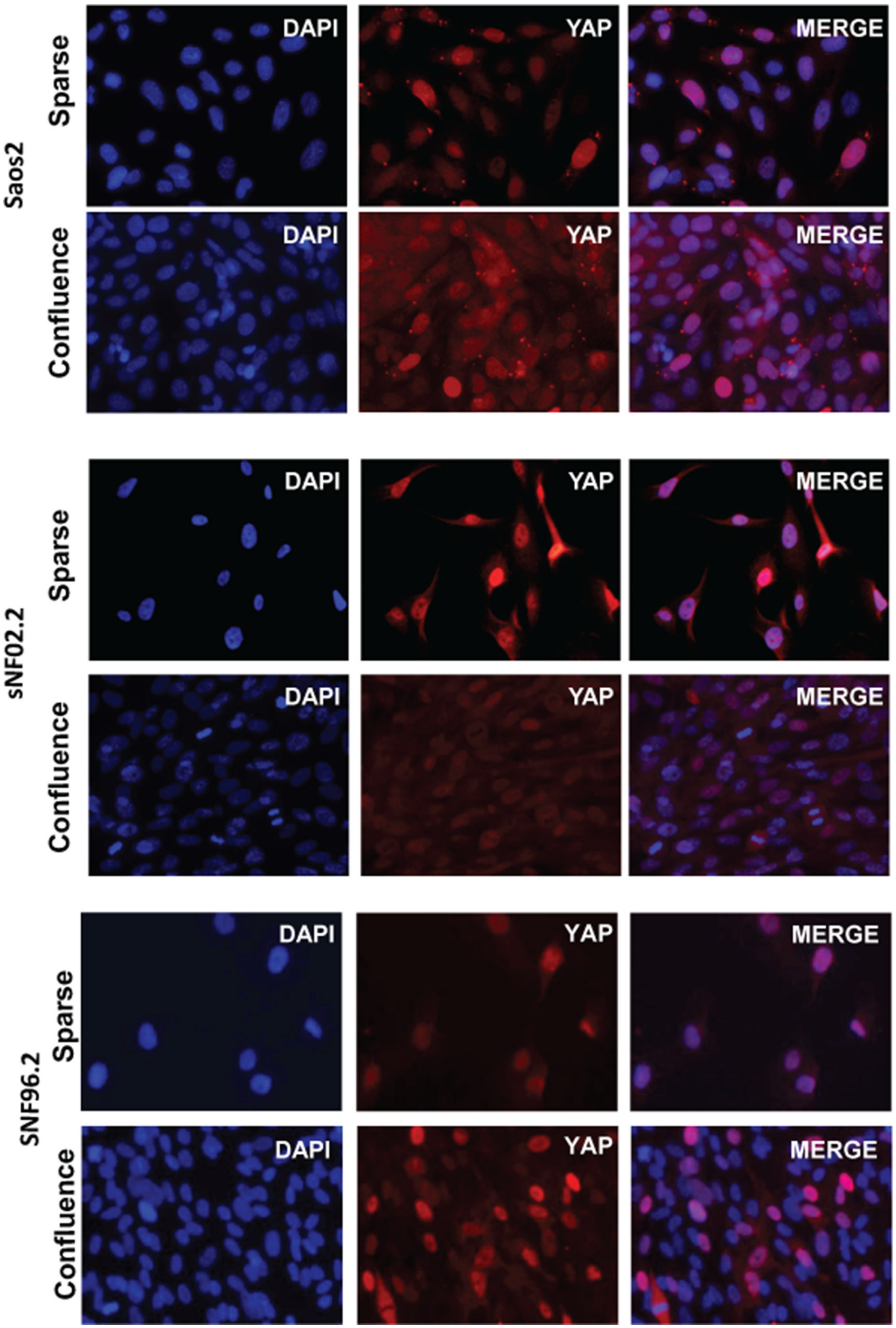
Saos2
Sparse
Confluence
DAPI
YAP
MERGE
sNF02.2
Sparse
Confluence
DAPI
YAP
MERGE
SNF96.2
Sparse
Confluence
DAPI
YAP
MERGE

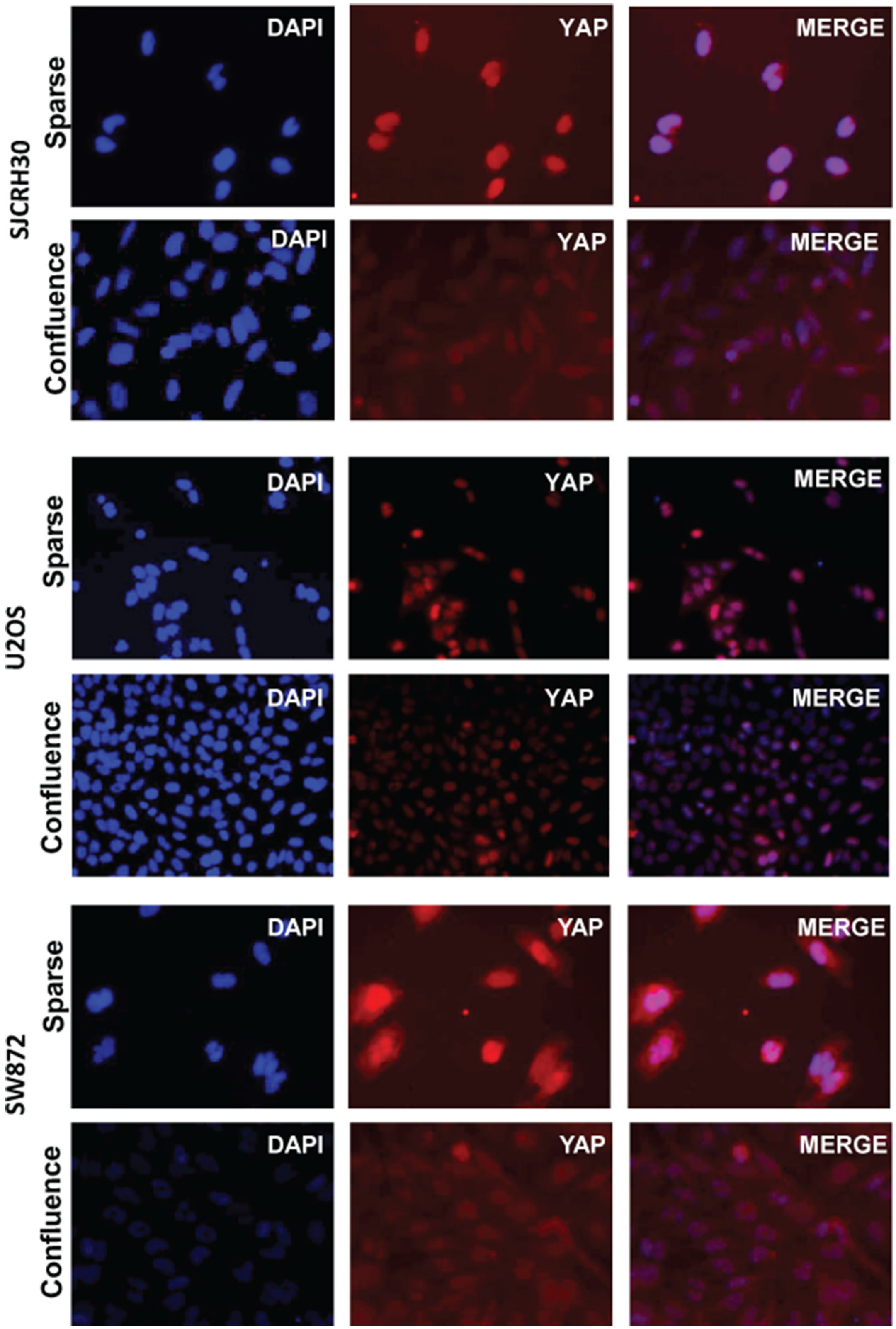

Supplementary Figure 3: Immunofluorescence for YAP during sparse and confluent conditions. GCT demonstrate diminished nuclear localization of YAP during confluent conditions, indicating the Hippo pathway is still regulating YAP in this cell line. The remaining sarcoma cell lines (HT1080, RD, SW982, A204, SW684, Saos2, SNF02.2, SNF96.2, SJCRH30, U2OS, and SW872) demonstrate YAP to be located predominantly within the nucleus.

A

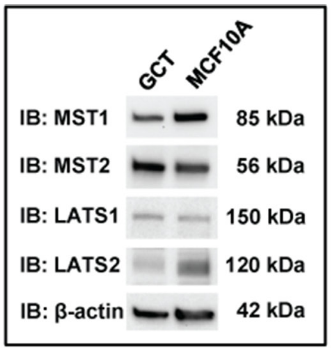

B

	***WWTR1* (TAZ)**		***YAP1* (YAP)**		
**Cell line**	Exon 2 (S66, S89, S117)	Exon 6 (S311)	Exon 1 (S61)	Exon 2 (S109, S127, S164)	Exon 7 (S381)
**GCT**	neg	neg	neg	neg	neg
**HT1080**	neg	neg	neg	neg	neg
**SKLMS1**	neg	neg	neg	neg	neg
**RD**	neg	neg	neg	neg	neg
**SW982**	neg	neg	neg	neg	neg
**A204**	neg	neg	neg	neg	neg
**SW684**	neg	neg	neg	neg	neg
**SAOS2**	neg	neg	neg	neg	neg
**SNF02.2**	neg	neg	neg	neg	neg
**SNF96.2**	neg	neg	neg	neg	neg
**SJCRH30**	neg	neg	neg	neg	neg
**U2OS**	neg	neg	neg	neg	neg
**SW872**	neg	neg	neg	neg	neg
**Total**	**0/13**	**0/13**	**0/13**	**0/13**	**0/13**

Supplementary Figure 4: (**A**) Western blot of the GCT and MCF10a cell lines, demonstrating similar expression of the Hippo kinases. (**B**) *WWTR1* and *YAP1* were Sanger sequenced for mutations involving serines phosphorylated by the Hippo pathway. This includes S66, S89, S117, and S311 of *WWTR1* and S61, S109, S127, S164, and S381 of *YAP1*. PCR primers were designed flanking two amplicons, exon 2 (S66, S89, S117) and exon 6 (S311) of *WWTR1*. For *YAP1*, PCR primers were designed that flanked exon 1 (S61), exon 2 (S109, S127, S164), and exon 7 (S381). No mutations were identified in either exon 2 or exon 6 of *WWTR1*, or exon 1, exon2, or exon 7 of *YAP1*.

A

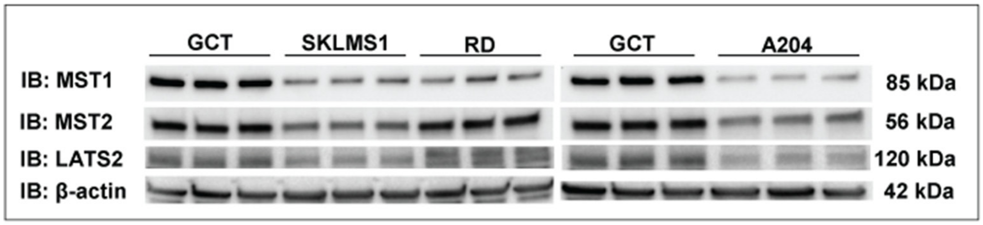

B

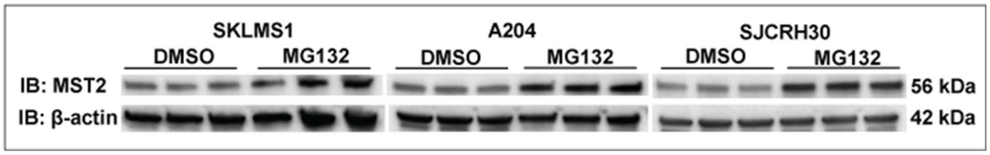

Supplementary Figure 5: Quantitative Western blots with samples run in triplicate. (**A**) Expression of MST1, MST2, and LATS2 evaluated in the SKLMS1, RD, and A204 cell lines. (**B**) Accumulation of MST2 expression with MG132 treatment in the SKLMS1, A204, and SJCRH30 cell lines.

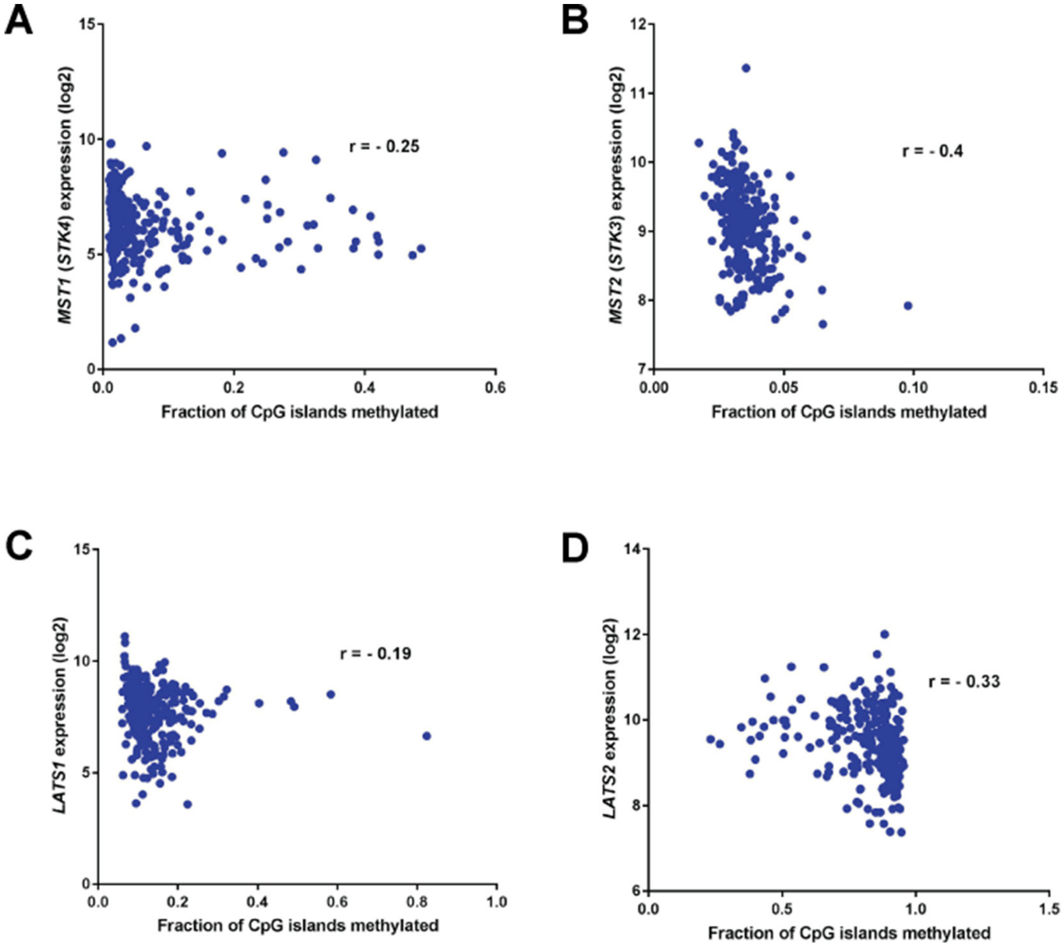

Supplementary Figure 6: Methylation of CpG islands correlated with RNA expression (RSEM [$\log_2$] for *MST1, MST2, LATS1,* and *LATS2* (**A**–**D**) demonstrating The Cancer Genome Atlas TCGA methylation data of 259 sarcomas acquired from Firebrowse.org. A modest correlation with CpG island methylation and RNA expression was identified for (B) *MST2* ($r = -0.4$, $p < 0.0001$). No significant correlation was identified for (A) *MST1* ($r = -0.25$), (C) *LATS1* ($r = -0.19$), and (D) *LATS2* ($r = -0.33$).

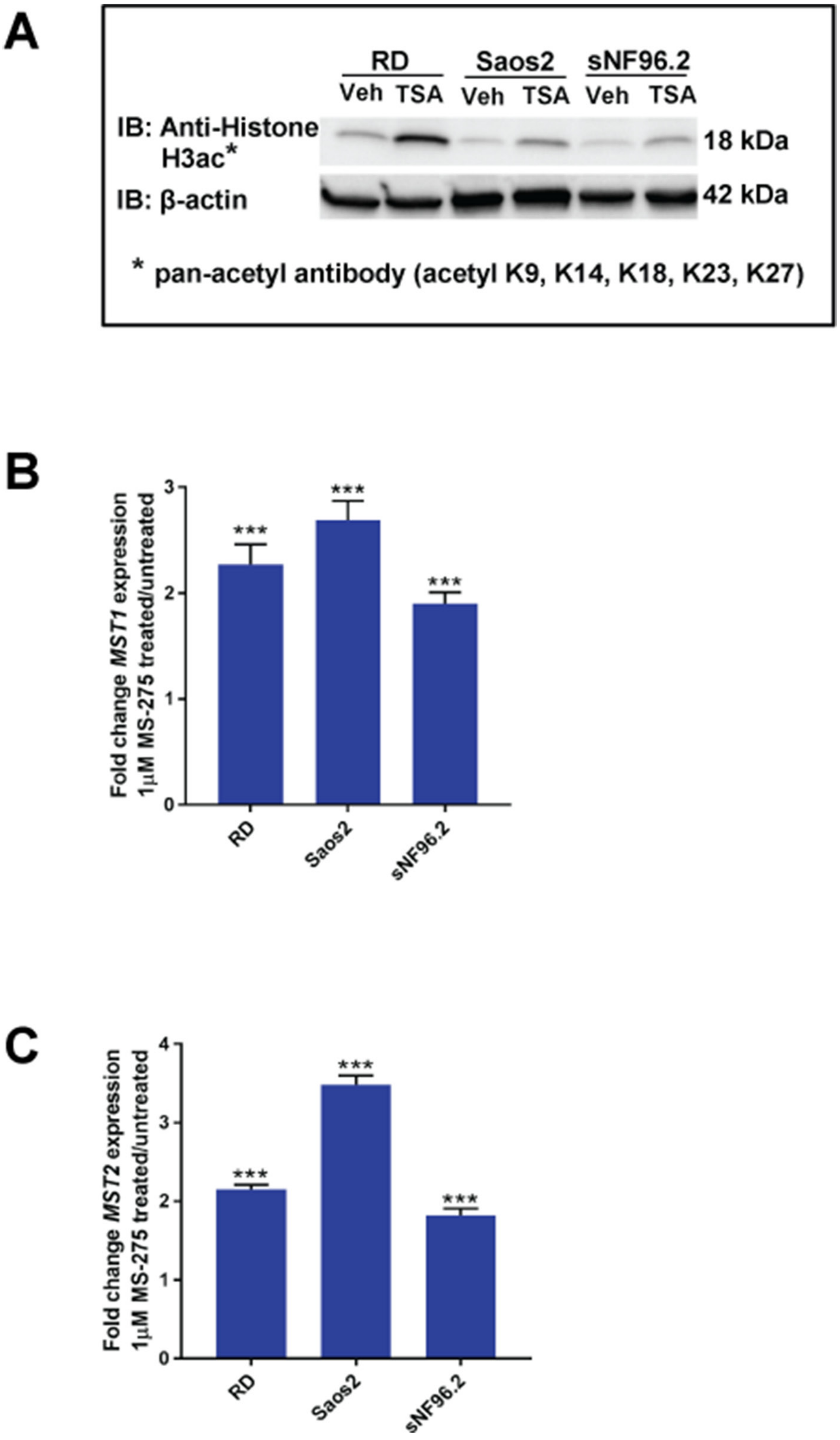

Supplementary Figure 7: (**A**) Treatment with 0.5 μM trichostatin A for 24h reveals an increase in acetylated Histone H3 in the RD, Saos2, and sNF96.2 cell lines. (**B**) Treatment with 1 μM MS-275 for 24 h demonstrated an at least 1.5 fold increase in *MST1* expression in the RD, Saos2, and sNF96.2 cell lines. (**C**) Treatment with 1 μM MS-275 for 24 h demonstrated an at least 1.5 fold increase in MST2 expression in the RD, Saos2, and sNF96.2 cell lines. Statistical significance determined by two-tailed *t*-test. * indicates $p < 0.05$; ** indicates $p < 0.01$; *** indicates $p < 0.001$.
